# Cryptic genetic diversity in the mottled rabbitfish *Siganus fuscescens* with mitochondrial introgression at a contact zone in the South China Sea

**DOI:** 10.1371/journal.pone.0193220

**Published:** 2018-02-21

**Authors:** Rachel Ravago-Gotanco, Talna Lorena de la Cruz, Ma. Josefa Pante, Philippe Borsa

**Affiliations:** 1 The Marine Science Institute, University of the Philippines Diliman, Quezon City, Philippines; 2 Institut de recherché pour le développement (IRD), “Ecologie marine tropicale des oceans Pacifique et Indien” c/o Indonesian Biodiversity Research Center, Universitas Udayana, Denpasar, Indonesia; National Cheng Kung University, TAIWAN

## Abstract

The taxonomy of the mottled rabbitfish *Siganus fuscescens* species complex has long been challenging. In this study, we analyzed microsatellite genotypes, mitochondrial lineages, and morphometric data from 373 *S*. *fuscescens* individuals sampled from the northern Philippines and Hong Kong (South China Sea, Philippine Sea and Sulu Sea basins), to examine putative species boundaries in samples comprising three co-occurring mitochondrial lineages previously reported to characterize *S*. *fuscescens* (Clade A and Clade B) or *S*. *canaliculatus* (Clade C). We report the existence of two cryptic species within *S*. *fuscescens* in the northeast region of the South China Sea and northern Philippine Sea, supported by genetic and morphological differences. Individual-based assignment methods recovered concordant groupings of individuals into two nuclear genotype clusters (Cluster 1, Cluster 2) with (1) limited gene flow, if any, between them (*F*_*ST*_ = 0.241; *P* < 0.001); (2) low frequency of later-generation hybrids; (3) significant association with mitochondrial Clade A and Clade B, respectively; and (4) subtle yet significant body shape differences as inferred from geometric morphometric analysis. The divergence between mitochondrial Clade C and the two other clades was not matched by genetic differences at microsatellite marker loci. The occurrence of discordant mitonuclear combinations (20.5% of the total number of individuals) is thought to result from mitochondrial introgression, consistent with a scenario of demographic, and presumably spatial, post-Pleistocene expansion of populations from northern regions into a secondary contact zone in the South China Sea. Mitonuclear discordance due to introgression obscures phylogenetic relationships for recently-diverged lineages, and cautions against the use of mitochondrial markers alone for species identification within the mottled rabbitfish species complex in the South China Sea region.

## Introduction

Molecular genetic approaches enhance our understanding of biodiversity patterns and mechanisms. The recognition of cryptic species has increased in frequency with the use of molecular genetic markers [1, 2]. Uncovering and characterizing spatial distribution patterns of cryptic diversity provide insight into the processes that influence diversification and speciation, particularly in the marine environment where few barriers to dispersal and gene flow are apparent [3–5]. In the Coral Triangle, the global hotspot of marine biodiversity [6, 7], contrasting phylogeographic patterns have been observed across taxa [8–11]. These results suggest that various biogeographic hypotheses proposed to explain the origins of the exceptional species richness in the region may not be mutually exclusive and may act in concert, underscoring the complexity of marine diversification mechanisms [5, 12, 13].

The family Siganidae, commonly known as rabbitfishes, consists of 29 recognized species, with greatest species richness observed in the Coral Triangle [14–16]. Although species groups are identified primarily based on body depth and coloration [14], some closely-related species remain difficult to distinguish because of a lack of sharp differences in color patterns [14]. In particular, the white-spotted rabbitfish *Siganus canaliculatus* (Park, 1797) may be difficult to differentiate from the mottled rabbitfish *Siganus fuscescens* (Houttuyn, 1782). The two species occur sympatrically across the central Indo-West Pacific [14], where they occupy similar seagrass and reef-associated habitats. They are primarily distinguished based on the size and number of white spots on the body, with *S*. *canaliculatus* having larger and fewer spots relative to *S*. *fuscescens* [14]. Genetic studies based on allozyme markers [17] and mitochondrial cytochrome *b* (cyt*b*) gene sequences [18, 19] have suggested the recognition of *S*. *fuscescens* and *S*. *canaliculatus* as valid biological species because sympatric populations of the two species exhibit significant allozyme frequency differences and are characterized by divergent mitochondrial lineages. This conclusion is challenged by the report of paraphyletism in both cyt*b* and internal transcribed spacer (ITS) sequence phylogenies of *S*. *canaliculatus* and *S*. *fuscescens* morphotypes [20], and by non-concordance between morphotype and genetic clustering based on AFLP loci [21]. Authors have concluded that *S*. *fuscescens* and *S*. *canaliculatus* interbreed, hence represent morphological variants or geographically isolated groups within a single biological species [20, 21].

Apart from the conflicting evidence regarding the species status of *S*. *canaliculatus* and *S*. *fuscescens*, additional cryptic diversity has been reported within the *S*. *fuscescens* morphotype. Allozyme data suggest two cryptic species of *S*. *fuscescens* across the broader Coral Triangle region [17]. Mitochondrial cyt*b* and control region (CR) phylogenies meanwhile reveal the sympatric occurrence of three divergent mitochondrial lineages of *S*. *fuscescens*, designated Clade A, Clade B and Clade C, in the Philippines, at the northern apex of the Coral Triangle, and a location further north (Hong Kong) [19]. Clade A is widely-distributed across the central Indo-West Pacific region, occurring in the Philippines, Japan, Taiwan, the Indonesian archipelago, the Solomon Islands and New Caledonia [18, 22]. The distribution of Clade B appears to be restricted to the northern periphery of the *S*. *fuscescens’* distributional range, having been reported from Japan, Taiwan, the northern Philippines, Vietnam and Thailand [21, 23–26]. Clade C is present in the northwestern Pacific [19–21, 23, 26]. Mitochondrial DNA sequences have been useful in the study of lineage diversification in Siganidae [18, 20], and of their phylogeography and population genetic structure [19, 21, 23, 26]. However, the mitochondrial marker alone is inadequate to detect reproductive isolation and species boundaries [27], and its utility to identify species is limited when there is cross-species hybridization and introgression [28]. Combining mitochondrial markers with nuclear markers is a powerful means to examine potential cryptic speciation and species boundaries within a species complex.

In this study, we use mitochondrial DNA sequences and nuclear (microsatellite) markers, to test the hypothesis of cryptic speciation among co-occurring lineages of the mottled rabbitfish species complex in the northern Philippines and Hong Kong. If the mtDNA lineages characterize reproductively isolated groups, we expect: (1) matching differences in the nuclear genome, in the form of distinct microsatellite genotypic clusters with minimal admixture [29]; and (2) mitonuclear disequilibrium, i.e. a significant association between mtDNA lineage and genotype cluster [30–32]. However, concordance between mitochondrial and nuclear patterns is not always observed among recently-diverged lineages [33, 34]. Mitonuclear discordance may be caused by incomplete lineage sorting of ancestral polymorphism, or recent gene flow or introgression between previously isolated lineages, e.g. secondary contact following allopatric divergence (reviewed in [35]). To evaluate these two competing hypotheses, we employ coalescent analysis under an isolation-with-migration (IM) model to jointly estimate mitochondrial gene flow and the timing of lineage divergence. In addition, geometric morphometric analysis enables us to characterize shape variation and examine the potential association between morphological variation and genetic differentiation.

Using an integrative approach combining genetic data from mitochondrial and nuclear markers with morphometric analysis, we demonstrate that *S*. *fuscescens* specimens from locations where three mitochondrial lineages occur in sympatry constitute two cryptic species. We also report mitonuclear discordance likely due to introgression, which cautions against the use of mitochondrial markers alone for species identification in the mottled rabbitfish species complex.

## Materials and methods

### Sample collection

**Fig 1:**
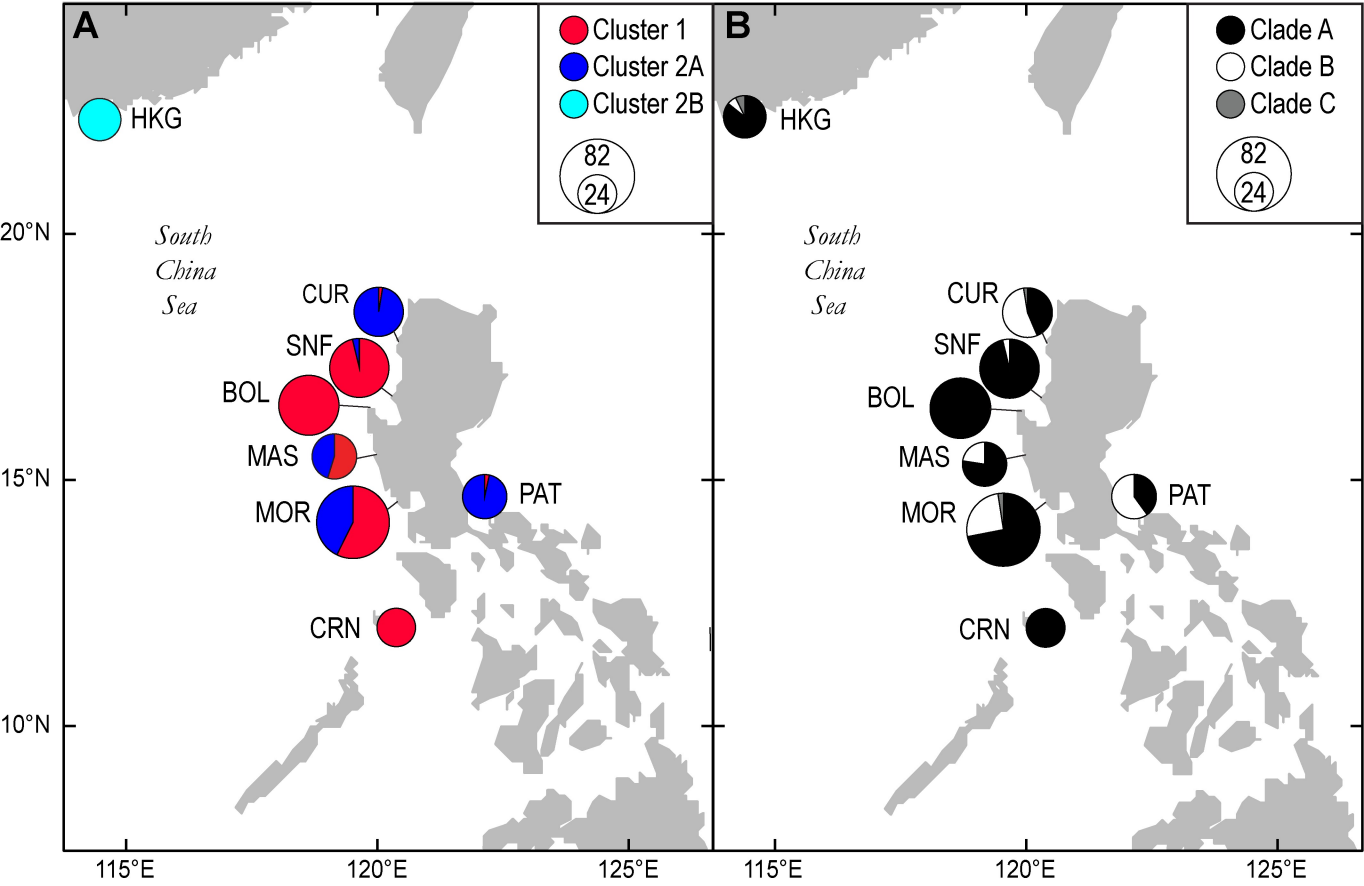
Distribution of *Siganus fuscescens* genotype clusters and mtDNA lineages across sampling locations in the South China Sea and Philippine Sea. Circle size is proportional to sample size. (A) Genotype clusters; (B) MtDNA clades. Background map of the northern Philippines and surrounding regions was downloaded from Digital Vector Maps, San Diego (http://digital-vector-maps.com/). Abbreviations for sampling locations: BOL, Bolinao; CRN, Coron; CUR, Currimao; HKG, Hong Kong; MAS, Masinloc; MOR, Morong; PAT, Patnanungan; SNF, San Fernando. (A) Genotype clusters; (B) MtDNA clades.

Mottled rabbitfish *Siganus fuscescens* specimens were collected from eight locations in the South China Sea, the Philippine Sea, and the Sulu Sea. Specimens from the Philippines were purchased from local fishermen who collected the samples within a 2–5 km radius from the collecting locality. These locations were Bolinao, Pangasinan (BOL); Coron, Palawan (CRN); Currimao, Ilocos Norte (CUR); Brgy. Sabang, Masinloc, Zambales (MAS); Morong, Bataan (MOR); Patnanungan, Polillo island, Quezon (PAT); and San Fernando, La Union (SNF) (Fig 1). Specimens from Hong Kong (sample HKG; Fig 1) were purchased from fishermen in the Sai Kung district. A small piece of muscle tissue was biopsied and preserved in 95% ethanol. DNA was extracted from tissue samples using a Chelex-proteinase K protocol [36]. A map showing the locations of the eight sampled populations is shown (Fig 1).

### Ethics statement

*Siganus fuscescens* is a commercially-harvested species in the Philippines and Hong Kong, and is not included in any IUCN or CITES list. All collections were performed by commercial fishers in public domains, and no permission was required for access.

### Microsatellite genotyping

Individuals were genotyped at 12 microsatellite markers previously developed for *S*. *fuscescens*: *Sfus6*, *Sfus8*, *Sfus9*, *Sfus21*, *Sfus22*, *Sfus56*, *Sfus76*, *Sfus95*, *Sfus97*, *Sfus98*, *Sfus113*, and *Sfus167* [24]. Microsatellite DNA fragments were PCR-amplified in four separate multiplex reactions. Each reaction consisted of 1x PCR Buffer, 2.0 mM MgCl_2_, 100 ng/μL bovine serum albumin, 200 μM of each dTNP, 0.25 units *Taq* polymerase (New England Biolabs, Ipswich MA), 0.25 μM each primer, and 1 μL template DNA in a final reaction volume of 10 μL. Forward primers were 5’-modified with 6-FAM^TM^, NED^TM^, PET^TM^, or VIC^®^ fluorescent dye. Amplification conditions consisted of 94°C for 90 s, 38 cycles of 94°C for 30 s, locus-specific annealing temperatures for 30 s, 72°C for 60 s and a final extension at 72°C for 25 minutes. Annealing temperatures, fluorescent label-primer combinations, and pooling of amplified microsatellite loci for fragment analysis have been described elsewhere [24]. Amplified fragments were combined with an internal size standard, GeneScan^TM^ 500 LIZ^TM^, Hi-Di^TM^ formamide (Applied Biosystems Inc., Carlsbad CA) and deionized water to a final volume of 10 μL. Fragments were run on an ABI 3730xl DNA Analyzer (Cornell University Life Sciences Core Laboratories Center, Ithaca NY). Allele calling was done in Geneious R6 (https://www.geneious.com) [37] using the Microsatellite plug-in. MICROCHECKER (http://www.microchecker.hull.ac.uk) [38] was used to check the microsatellite data for genotyping errors such as large allele drop-out, and to detect the possible presence of null alleles.

### Genotype cluster identification

Two approaches were used to identify genotype clusters from the microsatellite data. Correspondence analysis (CA), as implemented in the software GENETIX v. 4.05 (http://www.genetix.univ-montp2.fr/genetix/genetix.htm) [39] was run to visualize the distribution of individual microsatellite genotypes in multidimensional space, and identify clusters of individuals with similar genotypes. We also did Bayesian structure analysis using STRUCTURE v.2.3.4 (http://pritchardlab.stanford.edu/structure.html) [40], to assign individuals to clusters which minimize departures from Hardy-Weinberg equilibrium and linkage disequilibrium. Bayesian structure analysis also allows the estimation of the individual ancestry coefficient *q*, which is the proportion of an individual genotype having ancestry from each of *K* defined clusters. Ten replicate MCMC simulations were run for each value of *K* (*K* = 2 to 8). Each run comprised one million iterations, with an initial burn-in of 100,000 steps. An admixture model with correlated allele frequencies was chosen, using sampling location as a prior [41]. The most likely number of clusters was inferred based on the Δ*K* method [42], as implemented in the Structure Harvester tool (http://taylor0.biology.ucla.edu/structureHarvester/) [43]. Ancestry coefficients for each *K* value were averaged across replicate runs using CLUMPP [44]. To assign an individual to a cluster, we initially used a threshold value of *q* > 0.9 [45], while individuals having *q* values > 0.10 and < 0.9 were categorized as admixed. Results were plotted using DISTRUCT v. 1.1 [46].

To complement STRUCTURE results in identifying admixed individuals, we ran additional analyses using the program NEWHYBRIDS v. 1.1 [47]. NEWHYBRIDS assumes that the sample is drawn from a mixture of parental/purebred and admixed individuals, and estimates the posterior probability (*q*) that an individual belongs to one of six genotypic classes. In the present case, two parental classes corresponding to a scenario of *K* = 2 clusters (P1, P2), and 4 hybrid categories (F1, F2, F1 x P1 and F1 x P2) were initially assumed. Ten independent runs of 300,000 sweeps following a burn-in of 100,000 sweeps were done, with convergence assessed by visual examination of *P*(z) values from each run. Average NEWHYBRIDS *q* values were calculated from independent runs, and a probability threshold of *q* > 0.50 was used to assign individuals into classes.

### Mitochondrial lineage identification

The present paper focuses on cryptic speciation within *S*. *fuscescens* based on both mitochondrial and nuclear data. It includes mitochondrial control region (CR) sequences and subsequent lineage identification for 143 individuals derived from earlier work which examined the phylogeography and genetic connectivity of *Siganus fuscescens* across the Philippine archipelago [19]. To ascertain lineage membership for additional samples without DNA sequencing, a multiplex PCR approach was developed to selectively amplify clade-diagnostic CR fragments. Representative mtDNA control region sequences for mottled rabbitfish species complex Clade A, Clade B, and Clade C (S1 Table), were aligned and analysed using Geneious R6 [37] to identify clade-specific variable sites. Two clade-specific primers were designed using PRIMER3 [48]: a Clade C-specific forward primer (SigCR-141F,5’-TCAAAATAGCTTGGATTAAATACTGG-3’), and an *S*. *fuscescens* Clade B-specific reverse primer (SigCR-398R, 5’-CGATGAAAGATAGGGGAGTAA-3’). These were combined with primers 14F and 14R which specifically amplify the mt CR in *S*. *fuscescens* [49], in a multiplex PCR to amplify specific fragment sizes diagnostic for mtDNA lineages. PCR amplification was performed in 10 μL reaction volume comprising 1x PCR Buffer, 2 mM MgCl_2_, 200 μM of each dNTP, 0.25 units *Taq* polymerase (Invitrogen), 0.3 μM of each primer, and 1 μL template DNA. Cycling conditions were 94°C for 3 min, 35 cycles of denaturation at 94°C for 30 s, annealing at 65°C for 1 min, and elongation at 72°C for 2 min with a final extension at 72°C for 5 min. Amplified fragments were resolved on 2% TBE-agarose gels and detected following ethidium bromide staining, with fragment profiles diagnostic for mtDNA lineage (S1 Fig).

To test the null hypothesis of random association between *S*. *fuscescens* mitochondrial clades and the nuclear genotype clusters, we used the CNDm program [50]. This program estimates the normalized cytonuclear disequilibrium (D*), a measure of cytonuclear association, based on observed counts of mtDNA clade-microsatellite cluster combinations.

### Genetic diversity and differentiation

Microsatellite genotype and allele frequencies were used to obtain estimates of genetic diversity and differentiation for groups of individuals. To estimate diversity, we calculated the mean number of alleles, observed and expected heterozygosity (*H*_*O*_, *H*_*E*_) and allele frequencies using GENEPOP [51]. Allelic richness standardized for minimum sample size was calculated using the package *hierfstat* v. 0.04–22 for R [52]. Exact tests of deviations from Hardy-Weinberg equilibrium, the inbreeding coefficient (*F*_IS_), and exact tests of linkage disequilibrium between microsatellite loci were assessed using GENEPOP. To estimate levels of genetic differentiation we did an analysis of molecular variance (AMOVA) as implemented in ARLEQUIN v. 3.5 [53], to calculate hierarchical fixation indices, *F*_ST_ for different hypothesized groupings of individuals. We used *G"*_ST_ calculated using the GENALEX software package [54] as an unbiased estimator of *F'*_ST_, i.e. the value of *F*_ST_ standardized by the maximum obtainable value accounting for within-population heterozygosity [55, 56].

To visualize spatial patterns of genetic differentiation based on nuclear and mitochondrial markers, we used GENALEX for multidimensional clustering of populations using principal coordinates analysis (PCoA). We estimated population-pairwise *F*_ST_ and *Φ*_ST_ values calculated from microsatellite genotypes and mtDNA CR sequences, respectively. We tested for correlations between matrices of genetic distance (*F*_ST_ or *Φ*_ST_) and geographic distance using a Mantel test as implemented in the package IBDWS v. 3.2.3 (http://ibdws.sdsu.edu/~ibdws/) [57], with 1000 random permutations to test for significance.

### Estimating gene flow: Isolation-with-migration model

The Isolation-with-Migration model (IM) [58] was used to examine the relative contribution of competing demographic scenarios that explain the sharing of mitochondrial haplotypes between genotype clusters: (1) divergence under complete isolation (incomplete lineage sorting) and (2) divergence with gene flow (hybridization and introgression), by estimating the posterior probability of migration since the time of population splitting [59, 60]. We used the Bayesian Markov-Chain Monte Carlo method implemented in the program IMa2p [61], a parallel implementation of the IMa2 program [59], to jointly estimate posterior distributions of six demographic parameters, scaled by the mutation rate μ: population sizes for ancestral (q_2_) and descendant (q_0_, q_1_) populations, bidirectional migration rates (m_1>2_ and m_2>1_), where m is the migration rate per generation into the specified population, and divergence time (t) for pairs of populations. The IM model assumes that the populations under study are panmictic, markers are selectively neutral, and free from recombination. We tested the selective neutrality of nucleotide variation in CR sequences using Tajima's *D* [62], and Fu's *Fs* [63], estimated using ARLEQUIN. Tests for recombination were done on the CR sequence alignment using the RDP method [64] as implemented in the RDP3 package [65], and a pairwise homoplasy index (PHI) test [66]. None of these methods detected evidence of recombination in the control region sequence alignment. While IMa2p can analyse multiple populations, a large number of loci are required for better estimation, and require a well resolved, rooted phylogeny of populations as input [67]. Consequently, we performed multiple population pairwise analyses instead, with mtDNA data analysed as three separate pairwise comparisons in which individuals were assigned to three genetic groups according to the microsatellite data (see results). Preliminary runs with broad parameter intervals were performed to set the upper bounds for *q*, *t* and *m* priors for each pairwise analysis. Following identification of appropriate values for priors, analyses were run until stationarity was reached before genealogy sampling commenced. Three independent MCMC runs of 100 million generations (100,000 genealogies) were done for each pairwise analysis under the HKY model of evolution. Sampled genealogies were combined in an L-mode analysis to estimate demographic parameters. The mutation-scaled IMa2p parameter estimates were converted to demographic quantities: divergence time (t), effective population size (N), and population migration rates (2Nm). We used two mutation rates, 2% and 5% per million years, to cover the range of mutation rates approximated for the control region in bony fishes [68]. The mutation rates were converted to per locus rates by multiplying the fragment length in base pairs for demographic conversions [69]. Demographic quantities were estimated following the equations given in the IMa documentation (https://bio.cst.temple.edu/~hey/software/software.htm). To convert divergence time from generations to years, we estimated generation time following the formula *T =* (α + ω)/2 where α is the age at first reproduction and ω is the age at last reproduction [70]. Age at first reproduction for *S*. *fuscescens* is 5 to 7 months, or roughly 0.5 year [71]. Maximum age was calculated to be 5 years, using an age-at length model for *S*. *fuscescens* derived by Bellefleur [72], for a maximum reported length of 25 cm [71]. Average generation time was thus estimated to be 2.75 years.

### Morphological differentiation and geometric morphometric analysis

Morphological identification of the specimens as *S*. *fuscescens* followed Woodland [14]. The left side of each fish was photographed using a mounted digital camera. The dorsal, pelvic, anal and caudal fins of each fish were spread out and pinned in place prior to photo documentation. Landmarks were chosen in accordance with the guidelines of Zelditch et al. [73]. A total of 22 landmarks were selected for their capacity to represent overall body shape (S2 Fig). The Cartesian coordinates of each landmark were digitized using tpsDig2 v. 2.05 (http://life.bio.sunysb.edu/morph/) [74]. Geometric morphometric analyses were subsequently conducted using the PAleontological STatistics (PAST) software v.1.72 [75]. To eliminate the confounding effect of size differences, the digitized raw landmarks were transformed using the generalized least squares Procrustes superimposition [76, 77], by initially scaling landmark configurations of all specimens to unit size, then aligning all landmarks as closely as possible. Size-independent landmark variables were then subjected to several ordination methods. Multivariate analyses were run using two approaches to simplify descriptions of morphological differences among groups of individuals. Principal component analysis [78] was conducted without *a priori* information from genetic data. Canonical variates analyses (CVA), were run based on *a priori* information separately for (1) mitochondrial clade; and (2) nuclear cluster assignments. Thin-plate spline (TPS) deformation grids were used to visualize the differences between landmarks over the entire body shape, based on the interpolation function of landmark differences, taking all displacements of all landmarks relative to all others into account [73, 79]. Multivariate analysis of variance (MANOVA) and pairwise comparisons were done to test the null hypothesis of no morphological difference between groups.

## Results

Multi-locus microsatellite genotypes, mtDNA lineage assignment based on control region sequences (direct sequencing or multiplex PCR methods), and morphometric measurements were obtained for a total of 343 individuals identified as *Siganus fuscescens* from their color patterns.

### Microsatellite genotype clusters

All 12 microsatellite loci analyzed were polymorphic. No identical multilocus genotypes were observed. Further, there was no evidence for large allele dropout following analysis of the genotype data using MICROCHECKER.

**Fig 2:**
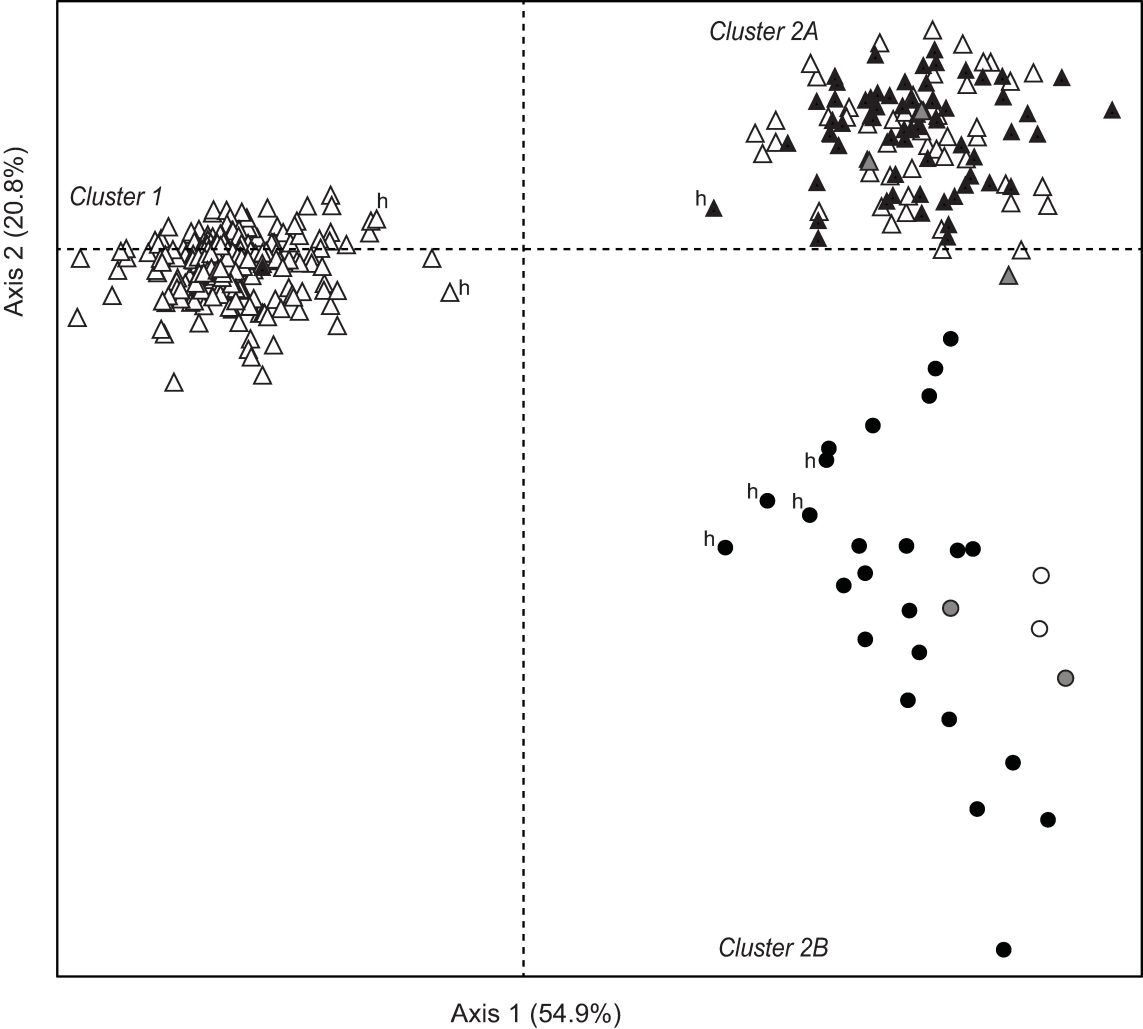
Siganus fuscescens. Two-dimensional plot of correspondence analysis of individual genotypes at 12 microsatellite loci. Each point represents an individual (total *N* = 343). Colors indicate mtDNA control region lineages: Clade A (black), Clade B (white), Clade C (grey). Symbols indicate sampling region: Philippines (triangles) and Hong Kong (circles). Putative hybrids as revealed by NEWHYBRIDS analysis are marked (‘h’).

Correspondence analysis (CA) of genotype data from 12 microsatellite loci clustered the 343 individuals into three distinct groups (Fig 2). Factorial axis 1 accounted for 54.9% of the total variation and differentiated a genotype cluster exclusive to the Philippines (hereafter Cluster 1) from a second cluster sampled on both sides of the South China Sea (Cluster 2). Factorial axis 2, which accounted for 20.8% of the total variation, separated individuals according to geographical location. Cluster 2 was split into two sub-clusters, one consisting of samples from the Philippines exclusively (a sub-cluster designated Cluster 2A), distinct from the other one, consisting of individuals collected from Hong Kong exclusively (designated Cluster 2B).

**Fig 3:**
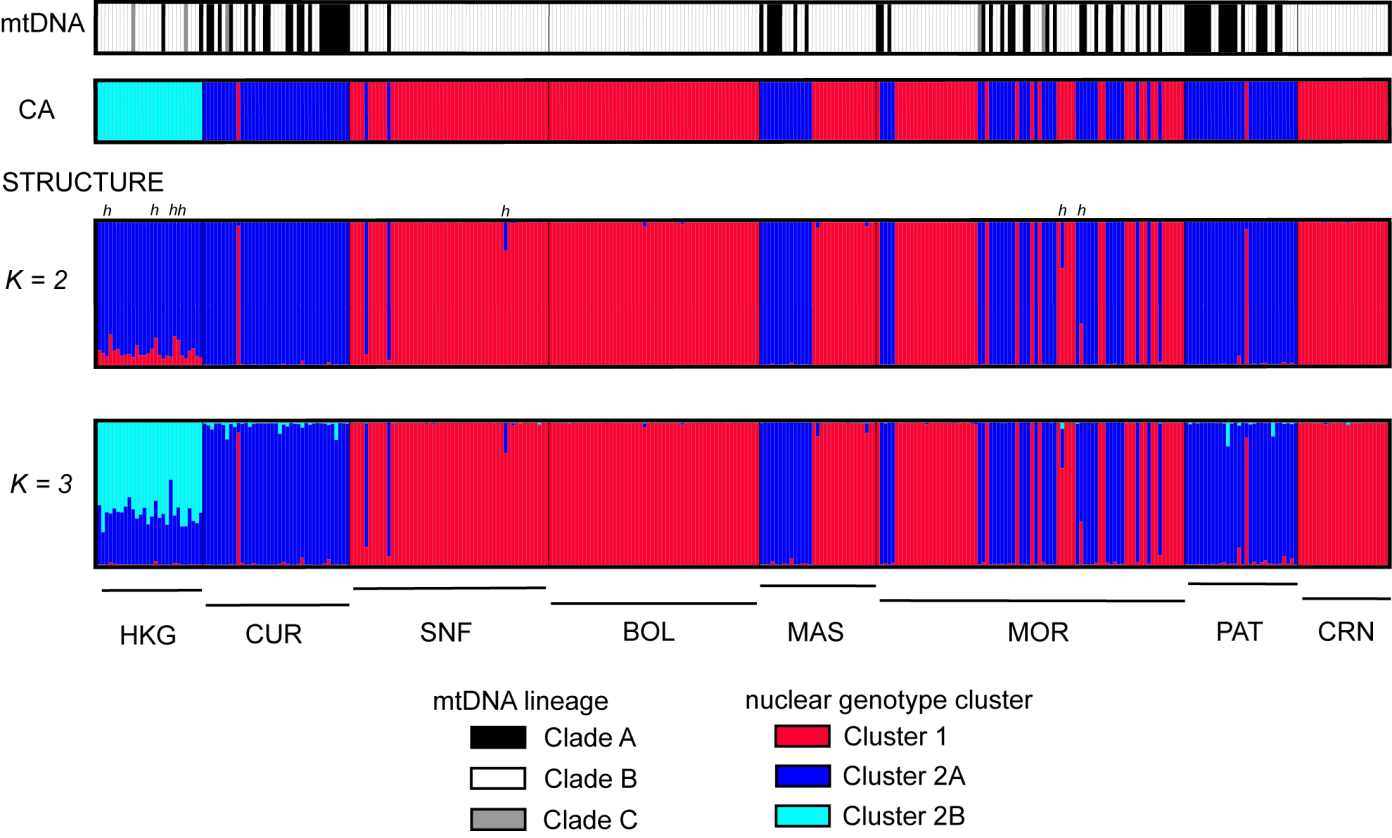
Comparison of genotype cluster assignment and mtDNA lineage membership for *Siganus fuscescens* individuals. Each individual is represented by a vertical bar where the apportion of ancestry is represented by segments of different colors corresponding to different mitochondrial lineages and nuclear genotype clusters. Individuals with mixed ancestry are indicated by different colors corresponding to the inferred proportion of ancestry in a particular group. (a) MtDNA lineage membership; (b) Assignment to nuclear genotype cluster based on correspondence analysis; (c) Individual ancestry coefficient (*q*) inferred from STRUCTURE analysis when *K* = 2; (d) Individual ancestry coefficient (*q*) inferred from STRUCTURE analysis when *K* = 3. Putative hybrids are marked (‘h’).

STRUCTURE results revealed the most likely number of clusters to be *K* = 2 based on the Δ*K* method. However, the highest posterior probability of the data was observed when *K* = 3, with individuals from Hong Kong exhibiting *q* values > 0.2 and < 0.8 (Fig 3) indicating admixture between two clusters. While the Δ*K* method detects the uppermost level of population structure, i.e. the lowest *K* value [42], it may underestimate the true number of clusters particularly when genetic differentiation is weak or complex, as in the case of isolation-by-distance [80]. To examine the possibility of further substructure, we performed additional STRUCTURE runs separately for Cluster 1 and Cluster 2 individuals. No further sub-structure was detected among Cluster 1 individuals. Further sub-structuring was detected among Cluster 2 individuals, with the HKG samples clearly emerging as a cluster distinct from its Philippine homologue (*q* > 0.9), consistent with the clustering uncovered using CA.

Under a scenario of *K* = 2 genotype clusters, STRUCTURE results indicate admixture (0.1 < *q* < 0.9) and potential hybrid ancestry for 13 individuals. The majority (*N* = 10) of the putative hybrids were in the HKG sample, with 1 and 2 putative hybrids in SNF and MOR, respectively. The NEWHYBRIDS algorithm however identified only 7 of the 13 individuals having mixed ancestry in STRUCTURE as putative hybrids. All 7 individuals (marked in Fig 3) were categorized as F2 hybrids: 4 from sample HKG, 1 from sample SNF, and 2 from sample MOR. The 5 HKG individuals identified as putative hybrids by STRUCTURE, but not by NEWHYBRIDS, were categorized as parental genotypes by NEWHYBRIDS.

### Mitochondrial lineage identification and distribution

For 143 individuals, mtDNA control region sequences (792 bp) were obtained from a previous study (S1 Table) [19]. The multiplex PCR approach was used for lineage identification of an additional 200 individuals. The majority of the haplotypes were identified as Clade A (77.8%), while Clade B constituted 20.7% of the total sample of haplotypes. Five individuals (1.5% of total) were characterized by Clade C haplotypes, previously reported to be *S*. *canaliculatus* [19]. Clade A and Clade B were sympatric in 6 of the 8 locations sampled, i.e. all locations except BOL and CRN (Table 1; Fig 1B). Clade C was sampled along with both Clades A and B from3 locations: HKG, MAS, MOR (Table 1; Fig 1B).

**Table 1:** Mitochondrial DNA lineage identification of *Siganus fuscescens* samples from the northern Philippines and Hong Kong.

Sample			Data type		MtDNA lineage		
Locality	Abbreviation	N	mtDNA	multiplex PCR	Clade A	Clade B	Clade C
Hong Kong	HKG	28	28	-	24	2	2
Currimao	CUR	39	20	19	17	21	1
San Fernando	SNF	53	-	53	51	2	-
Bolinao	BOL	56	24	32	56	-	-
Masinloc	MAS	31	-	31	24	7	-
Morong	MOR	82	22	60	59	21	2
Patnanungan	PAT	30	25	5	12	18	-
Coron	CRN	24	24	-	24	-	-
**Total**		343	143	200	267	71	5

Individuals sampled per population (*N*) were assigned to one of three previously reported mitochondrial clades in the mottled rabbitfish species complex, designated Clade A, Clade B, and Clade C [19], based on phylogenetic analysis of mitochondrial DNA control region sequence (mtDNA) or multiplex PCR approach (multiplex PCR).

### Discordance between mtDNA haplotype and microsatellite genotype

Cluster 1 of CA consisted almost entirely of individuals with Clade A haplotypes, with the exception of one individual possessing a Clade B haplotype, out of 197 individuals in the cluster. Clusters 2A and 2B combined consisted of a mix of individuals with different mtDNA lineages: 69 individuals with Clade A, 67 with Clade B, and 5 with Clade C. Factorial axis 2 separated individuals according to geographical location. The five individuals exhibiting Clade-C haplotypes were all in Cluster 2, and occurred in both sub-clusters 2A and 2B.

A large proportion of the total variance in microsatellite genotypes was explained by the partition of the total sample into two major genotype clusters, Cluster 1 and Cluster 2. This was evident from two clustering approaches (CA and STRUCTURE) where assignment of individuals to either cluster was consistent (Fig 3) and appeared to be broadly concordant with mtDNA variation.

Results from the test of cytonuclear equilibrium indicated a significantly positive association between mitochondrial lineage and genotype cluster. Clade-A haplotypes were generally associated with Cluster 1 (D* = 0.976, *P* < 0.0001), and Clade-B haplotypes were generally associated with Cluster 2 (D* = 0.953, *P* < 0.0001) (Table 2). These mitonuclear combinations accounted for 79.5% of the total sample size (individuals with mixed ancestry and individuals harboring mitochondrial Clade C being excluded from the total). The majority of the non-concordant ('mismatch') mitonuclear combinations were Clade A x Cluster 2. This concerned 67 out of 68 individuals (98%) (Table 3), all scored in locations where mitochondrial clades A and B were sympatric. The proportion of mismatch per location varied widely, but it was notably high in sample HKG (Table 3).

**Table 2:** Count of individual mitonuclear combinations for three mtDNA lineages and three nuclear genotypes (Cluster 1, Cluster 2, and admixed^a^) in *Siganus fuscescens* samples from the northern Philippines and Hong Kong.

MtDNA lineage	Nuclear genotype			
	Cluster 1	Admixed	Cluster 2	Total
Clade A	194	12	61	267
Clade B	1	1	69	71
Clade C	-	-	5	5
Total	195	13	135	343

^a^ Assignment to genotype cluster was based on STRUCTURE analysis [44] with K = 2 clusters. An individual was assigned to a cluster when the coancestry coefficient was *q* > 0.90 or *q* < 0.10, and identified as having admixed ancestry when 0.10 < *q* < 0.90.

**Table 3:** Geographical distribution of the number of mitonuclear combinations in *Siganus fuscescens* from the northern Philippines and Hong Kong.

Sample	Mitonuclear combination				Discordance (%)
	Clade A x	Clade B x	Clade A x	Clade B x	
	Cluster 1	Cluster 2	Cluster 2	Cluster 1	
HKG	0	2	20	0	90.9
CUR	1	21	16	0	42.1
SNF	50	2	0	0	0
BOL	56	0	0	0	0
MAS	17	7	7	0	22.6
MOR	45	19	13	1	18.0
PAT	1	18	11	0	36.7
CRN	24	0	0	0	0
**Total**	194	69	67	1	20.5

Assignment to genotype cluster was based on STRUCTURE [44] when the number of clusters K = 2. An individual was assigned to a cluster when coancestry coefficient *q >*0.50. MtDNA clade- nuclear genotype cluster combinations exhibiting significantly positive associations (i.e. Clade A x Cluster 1 and Clade B x Cluster 2) are designated as concordant, with the other combinations considered as discordant.

### Genotype clusters: Genetic diversity and differentiation

A wide range of allelic diversity was scored at the 12 microsatellite loci, from 4 to 40 alleles per locus (Table 4 and S2 Table). Significant deviations from Hardy-Weinberg equilibrium were detected at 42 of 96 population-locus tests (43.75%, S2 Table) and 7 loci overall, all with significantly positive *F*_*IS*_ values suggesting heterozygote deficit (S2 Table). Exact tests of linkage disequilibrium also revealed significant association for 19 locus pairs out of a total of 66 tests (66.3%). Since most samples consisted of a mixture of individuals from two genetically distinct clusters, the significant *F*_*IS*_ and linkage disequilibrium are explained, at least partly, by the Wahlund effect.

**Table 4:** Genetic diversity estimates at 12 microsatellite loci for three genotype clusters of *Siganus fuscescens* from the northern Philippines and Hong Kong.

Locus	Cluster 1						Cluster 2A						Cluster 2B					
	*N*	*A*_*R*_	*Ho*	*He*	*Fis*	*P*	*N*	*A*_*R*_	*Ho*	*He*	*Fis*	*P*	*N*	*A*_*R*_	*Ho*	*He*	*Fis*	*P*
*Sfus6*	195	5.9	0.508	0.640	0.210*	0.002	117	8.1	0.684	0.760	0.114*	0.000	29	10.0	0.655	0.858	0.240*	0.003
*Sfus8*	197	15.6	0.868	0.914	0.053	0.096	117	13.9	0.897	0.911	0.012	0.864	29	16.0	0.966	0.927	0.042	0.884
*Sfus9*	197	4.9	0.695	0.686	-0.012	0.103	117	4.1	0.504	0.588	0.195	0.018	29	4.0	0.414	0.442	0.065	0.671
*Sfus21*	197	15.8	0.883	0.921	0.043	0.022	117	10.3	0.632	0.695	0.093*	0.000	29	12.0	0.655	0.835	0.218*	0.002
*Sfus22*	197	13.0	0.817	0.841	0.031	0.753	117	5.6	0.154	0.170	0.225	0.163	29	4.0	0.552	0.569	0.031	0.751
*Sfus56*	197	1.9	0.056	0.064	0.128	0.185	117	3.4	0.444	0.525	0.213*	0.007	29	3.0	0.345	0.440	0.219	0.010
*Sfus76*	195	15.9	0.733	0.909	0.196	0.000	117	16.4	0.863	0.897	0.047	0.829	29	19.0	0.759	0.946	0.201	0.011
*Sfus95*	197	13.5	0.792	0.840	0.060	0.126	114	7.8	0.482	0.573	0.177	0.012	29	7.0	0.448	0.797	0.442*	0.000
*Sfus97*	197	2.3	0.127	0.119	-0.065	1	116	10.0	0.741	0.840	0.136*	0.003	29	8.0	0.758	0.678	0.122	0.598
*Sfus98*	197	16.2	0.898	0.914	0.020	0.493	117	12.3	0.709	0.855	0.165*	0.002	29	6.0	0.758	0.916	0.174*	0.001
*Sfus113*	196	21.1	0.806	0.941	0.146*	0.000	117	14.1	0.632	0.792	0.199*	0.000	29	11.0	0.621	0.831	0.257	0.013
*Sfus167*	197	2.7	0.142	0.143	0.006	0.696	117	4.0	0.333	0.324	0.094	0.712	29	5.0	0.655	0.717	0.088	0.873
Overall	196.6	10.7	0.611	0.661			.	9.2	0.590	0.661			29	9.6	0.632	0.746		

Sample size (*N*), Allelic richness (*A*_*R*_), observed heterozygosity (*Ho*), expected heterozygosity (*He*), fixation index (*F*_*IS*_) and its associated probability are indicated for each population and cluster. Significant departures from Hardy-Weinberg equilibrium expectations (*F*_*IS*_> or *F*_*IS*_< 0) are indicated by an asterisk, following a sequential Bonferroni adjustment of the significance threshold [81].

Grouping individuals into three genotype clusters, significant departures from Hardy-Weinberg equilibrium expectations were observed for 11 out of a total of 36 tests (30.56%), and overall at 3, 6 and 4 loci in Cluster 1, Cluster 2A, and Cluster 2B, respectively, with significantly positive *F*_*IS*_ values in all cases (Table 4). No significant association between pairs of loci was observed. Allelic richness was generally higher for Cluster 1 (10.7), compared to Cluster 2A and Cluster 2B (9.2 and 9.6, respectively). Relatively large differences in gene diversity at loci: *Sfus 22*, *Sfus 56*, *Sfus97*, *Sfus167* were observed among the three clusters (Table 4).

Substantial genetic difference was found between Cluster 1 and Cluster 2 (overall *F*_ST_ = 0.241, *P* < 0.001; *G"st* = 0.749), with all population-pairwise comparisons between the two clusters reaching significant *F*_ST_ values (S3 Table). All 12 loci exhibited significant differentiation between clusters, with the higher levels of differentiation observed at loci *Sfus56*, *Sfus167*, *Sfus22*, and *Sfus97* (Table 5). Nine and 11 loci exhibited private alleles for Cluster 1 and Cluster 2, respectively. The proportion of private alleles was high in 10 out of 12 loci (44% - 87% of the total number of alleles). However, there was no fixation for alternative alleles at any locus.

**Table 5:** Estimates of genetic differentiation and distribution of allelic diversity at 12 microsatellite loci in *Siganus fuscescens* from the northern Philippines and Hong Kong.

Locus	Population differentiation estimator			N private alleles^a^		N shared alleles^b^	N alleles^c^
	*F*_*ST*_ ^d^	*G*_*ST*_ ^e^	*G"*_*ST*_ ^f^	Cluster1	Cluster2		
*Sfus6*	0.170	0.091	0.598	-	6	7	13
*Sfus8*	0.005	0.030	0.055	3	-	19	22
*Sfus9*	0.274	0.475	0.749	3	1	5	9
*Sfus21*	0.124	0.037	0.730	6	3	15	24
*Sfus22*	0.409	0.067	0.970	13	2	7	22
*Sfus56*	0.676	0.055	0.907	-	2	2	4
*Sfus76*	0.077	0.160	0.806	4	7	22	33
*Sfus95*	0.055	0.227	0.212	11	3	8	22
*Sfus97*	0.403	0.273	0.706	-	13	2	15
*Sfus98*	0.059	0.029	0.554	8	4	15	27
*Sfus113*	0.104	0.002	0.944	14	6	17	37
*Sfus167*	0.662	0.471	0.937	2	3	3	8

^a^Number of private alleles, exclusive to each genotype cluster

^b^ Number of alleles shared between clusters

^c^ Total number of alleles

^d^ standardized variance in allele frequency calculated using Arlequin [53]

^e^ *G*_*ST*_ after Nei (1987) [82]

^f^ Unbiased estimator of *F’*_*ST*_ [56].

Genotype clusters and mitochondrial lineages exhibited contrasting patterns of genetic differentiation. Allele frequencies were not significantly different among Cluster 1 samples from five locations: BOL, CRN, MAS, MOR, and SNF, (*F*_ST_ = 0.002; *P* > 0.05). This apparent lack of genetic structure contrasts with the genetic differentiation observed for Clade A haplotype samples from BOL, CRN and MOR (*Φ*_ST_ = 0.090; *P* < 0.0001), with MOR significantly different from BOL and CRN (S3B Fig). Meanwhile, Cluster 2 samples from five sites (CUR, HKG, MAS, MOR, and PAT) exhibited genetic differentiation (*F*_ST_ = 0.047, *P <* 0.0001) and grouped into three populations concordant with geographical location: western Philippine population (including samples from CUR, MAS, MOR); the Philippine Sea population (represented by sample PAT) and the Hong Kong population (HKG) (S3A Fig). However, Clade B haplotype samples from four of these sites (CUR, HKG, MOR, PAT) did not exhibit significant differentiation in haplotype distribution (*Φ*_ST_ = 0.042; *P* = 0.123; S3B Fig). Furthermore, for Cluster 2 samples (and only these), the standardized genetic differentiation [*F*_ST_/(1-*F*_ST_)] was apparently correlated with geographical distance (Mantel test: Z = 3544.3, *r* = 0.936, *P* = 0.016) (S4 Fig), suggesting possible isolation by distance.

### Gene flow estimates

**Fig 4:**
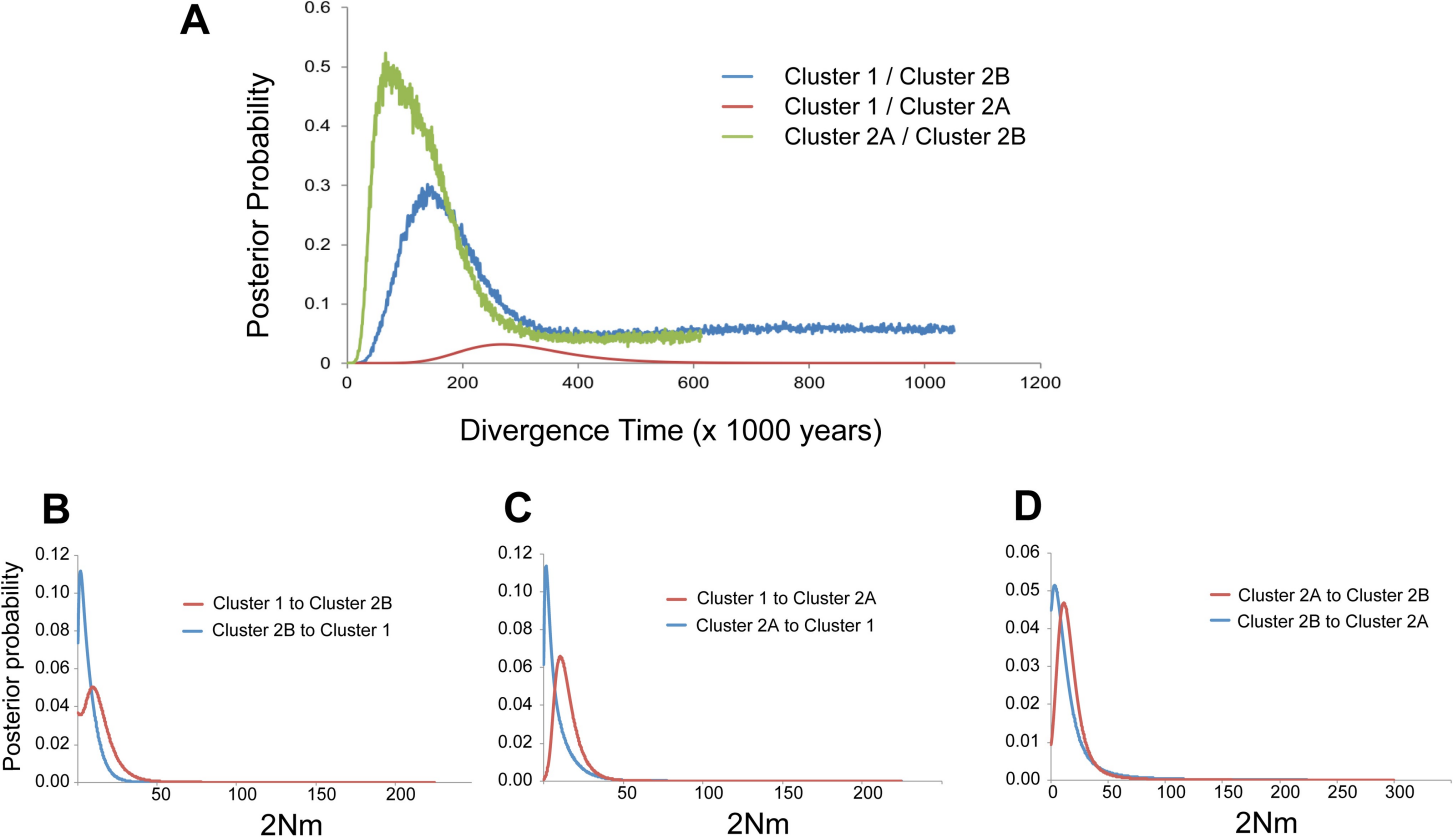
Posterior probability densities for divergence time and migration rate for *Siganus fuscescens* mitochondrial lineages within each of three nuclear clusters. Probability densities were estimated under the isolation-with-migration model (IMa2; [63]). (a) Estimated divergence times; (b, c, d). Pairwise estimates of population migration rate (2Nm) between nuclear clusters.

The IMa2p analyses of mtDNA sequences suggested recent divergence of the three genotype clusters, with asymmetrical migration between clusters. Based on mutation rates of 2% and 5% substitution per million years for the control region, the divergence between pairs Cluster 1/Cluster 2A and Cluster 1/Cluster 2B was estimated to have occurred approximately 111,000–298,000 years before present (BP) (Table 6), with the 95% highest posterior density (HPD) between 33,000–820,000 years BP. The divergence between Cluster 2A and Cluster 2B was more recent, between 53,000 to 133,000 years BP (95% HPD = 21,000–290,000 years BP). All posterior probability plots for divergence time showed well-defined unimodal peaks (Fig 4), although the distribution for Cluster 1/Cluster 2A was relatively broader.

**Table 6:** Isolation-with-migration analysis of mtDNA control region sequences for three pairwise comparisons of nuclear genotype clusters (Cluster 1, Cluster 2A, Cluster 2B) in *Siganus fuscescens* from the northern Philippines and Hong Kong.

Parameter	Pairwise comparisons		
Population 1	Cluster 1	Cluster 1	Cluster 2A
Population 2	Cluster 2A	Cluster 2B	Cluster 2B
Substitution rate = 2%			
Divergence time (generations)	108,153	101,274	48,375
N_1_	698,726	610,000	686,305
N_2_	517,197	579,000	708,598
N_A_	335,509	321,000	396,496
			
Substitution rate = 5%			
Divergence time (generations)	40,509	43,261	19,350
N_1_	244,000	279,490	274,522
N_2_	232,000	206,878	283,439
N_A_	129,000	134,203	158,598
			
Population migration rate			
2Nm_1_	1.65	1.47	3.22
2Nm_2_	9.43*	10.49*	11.21*
2Nm_1_ HPD95 Low—High	0.01–27.32	0.01–18.13	0.03–59.22
2Nm_2_ HPD95 Low -High	0.07–30.86	0.09–31.15	0.10–42.43

The effective population size of ancestral (N_A_) and descendant populations (N_1_, N_2_) were estimated based on two substitution rate values (2% and 5% substitutions per My). Population migration rates into each population (2Nm_1_, 2Nm_2_), and the 95% highest posterior density values (HPD95 Low–High) are indicated. Significant (*P* < 0.05) migration rates based on log likelihood ratio tests (LLR) are marked with an asterisk (*)

Asymmetrical mitochondrial gene flow occurred during divergence of the two major genotype clusters, Cluster 1 and Cluster 2. The population migration rate (2Nm) was significantly non-zero from Cluster 1 to Cluster 2A (2Nm = 10.4; LLR *P* < 0.05; Table 6), where the estimated probability of zero migration was also zero (Fig 4). However, migration in the opposite direction (Cluster 2A to Cluster 1) was not significantly higher than zero (2Nm = 1.47; LLR: *P* > 0.05; Table 6, Fig 4). Bidirectional migration rates between Cluster 1 and Cluster 2B were likewise asymmetrical, with gene flow from Cluster 1 to Cluster 2B (2Nm = 9.44, LLR: *P* < 0.05; Table 6) higher than gene flow in the opposite direction (2Nm = 1.65; LLR: *P* > 0.05). Similar patterns of asymmetrical gene flow occurred between Cluster 2A and Cluster 2B, with migration rate into Cluster 2B significantly non-zero (2Nm = 11.21; Table 6), and higher than migration into Cluster 2A (Fig 4).

### Geometric morphometric analysis

**Fig 5:**
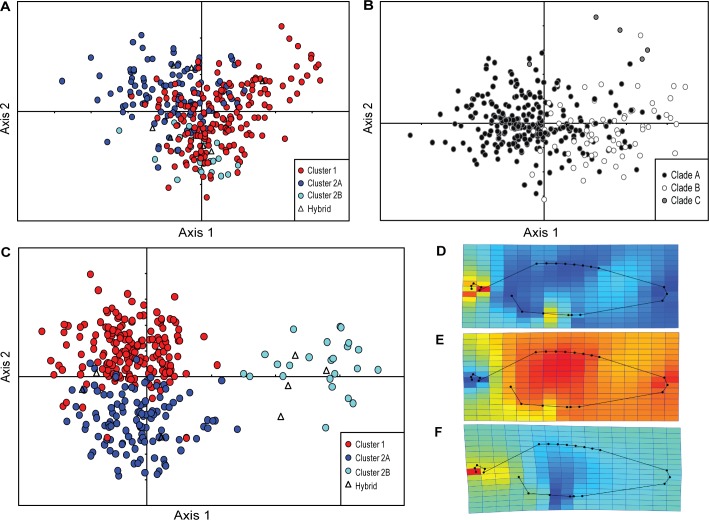
Scatter plot of shape differences in *Siganus fuscescens* following geometric morphometric analysis. Each point represents an individual. Circle color indicates nuclear genotype cluster or mtDNA lineage. Scatter plot of individual scores based on analysis of 22 landmarks: (A) Principal plan (PC1 x PC2) of principal components analysis where individuals are distinguished according to nuclear genotype cluster; (B) Principal plan (CV1 x CV2) of canonical variates analysis where individuals are distinguished according to mtDNA lineage; (C) Principal plan of canonical variates analysis where individuals are distinguished according to nuclear genotype clusters; (D) Thin-plate spline deformation grid for the average Cluster 1 individual relative to the consensus from the total sample; pixel color ranges show compression (dark blue) to expansion (red); (E) Thin-plate spline deformation grid for the average Cluster 2A individual; (F) Thin-plate spline deformation grid for the average Cluster 2B individual.

The first 3 principal components (PC) accounted for 69.5% of the total variation in shape among *S*. *fuscescens* specimens. PC1 represents 36.21% of total variance, and may be considered to be the major shape axis, while PC2 represents 23.53% of total variance. Ordination of PC1 and PC2 showed large overlaps between groups for microsatellite genotypes (Fig 5A) and mtDNA clades. This was due to a combination of highly negative loadings of landmarks on the dorsal fin (landmarks 6–13) and highly positive loadings of landmarks on the anal fin (landmarks 17–19), with moderate loading contribution from landmarks on the snout (landmark 1–5) and the pectoral–pelvic area (landmarks 20–22).

Canonical variates analysis (CVA) based on nuclear genotype cluster membership clustered individuals into generally non-overlapping morphospaces (Fig 5C). Meanwhile, CVA based on mtDNA lineage showed broad overlaps between Clade A and Clade B morphospaces, and Clade C individuals grouped with either Clade A or Clade B individuals (Fig 5B). Multiple Analysis of Variance (MANOVA) revealed significant differences in body shape among the 22 landmarks for both the microsatellite clusters and mitochondrial clades.

Pairwise comparisons of mitochondrial clades A, B and C, revealed significant difference between Clades A and B only (Wilk’s lambda = 0.523; Pillai trace = 0.537; *P* < 0.0001). Meanwhile, all pairwise comparisons between microsatellite Clusters 1, 2A, and 2B were significant (Wilk’s lambda = 0.114; Pillai trace = 1.320; *P* < 0.0001).

Thin-plate spline transformation grids indicate that Cluster 1 individuals were characterized by an expansion of the snout region (landmarks 1–5) and a slight contraction of the overall body area, particularly the dorsal, pectoral-pelvic and caudal areas (landmarks 6–16, 21–22), relative to the average shape (Fig 5D). Cluster 2A individuals were characterized by a contraction of the snout area (landmarks 1–5) and an overall expansion of the body and the caudal region (Fig 5E). Cluster 2B individuals were characterized by an expansion of the snout area and a contraction of the pelvic-anal area (Fig 5F).

## Discussion

This study revealed some incongruence in the patterns of genetic variation and population structure between mtDNA and microsatellite markers, both highlighting the complex evolutionary and demographic history of the *S*. *fuscescens* species complex and providing novel insights into it.

### Cryptic speciation in *S*. *fuscescens*: Limited gene flow and associated morphological differences between sympatric genotypic clusters

*Siganus fuscescens* individuals grouped into two main nuclear genotype clusters, with limited gene flow between them. The sympatric occurrence of differentiated nuclear genotypic clusters indicates reproductive isolation, hence cryptic species. Grouping individuals into two clusters accounted for a large proportion of the total genetic variation, and individual assignment to genotype cluster was robust and concordant between two different clustering approaches, i.e. correspondence analysis (CA), and Bayesian assignment. The signal of three genotype clusters recovered through all three approaches was due to the genetic partition of Cluster 2 into two sub-clusters. One of these two sub-clusters (Cluster 2A) was exclusive to the Philippines, and the other one (Cluster 2B) exclusive to Hong Kong. This sub-clustering was consistent with an isolation-by-distance scenario of genetic differentiation. Therefore, there is so far no reason to suspect additional cryptic speciation within Cluster 2.

Limited gene flow between genotype clusters is indicated by significant genetic differentiation between Cluster 1 and Cluster 2 at all 12 microsatellite marker loci. Microsatellite- or intron-based estimates of genetic differentiation of *F*_ST_ (or equivalent) > 0.200, as is the case here between *S*. *fuscescens* Clusters 1 and 2, may well indicate separate species (see [83–85]). For example, microsatellite-based estimates of genetic differentiation were as low as *F*_ST_ = 0.029 between sibling species of the humbug damselfish, *Dascyllus abudafur* and *D*. *aruanus* [86, 87], *F*_ST_ = 0.041 between cryptic species of the European anchovy *Engraulis encrasicolus* [88] and *F*_ST_ = 0.060 between reproductively isolated species within the grey mullet *Mugil cephalus* [89]. In *S*. *fuscescens*, the low proportion of putative F2 hybrids (< 4% of the total number of samples, or ~ 2% following a more stringent criterion for the identification of hybrids), provides further support to limited contemporary interbreeding between genotype clusters. This result is unlikely to be due to a low power to detect hybrids, i.e. type II error identifying true hybrids as parental genotypes. Both the STRUCTURE and NEWHYBRIDS approaches are expected to be highly efficient, with > 95% power to detect an individual's true status given the combination of the number of loci used in the present study and the observed level of differentiation as explained in the following. Simulations indicate that 12 polymorphic microsatellite loci is an adequate number of markers to detect hybrid status for a lower threshold *F*_ST_ = 0.210 between species [45]. Meanwhile, the discrepancy in the number of putative hybrids identified by STRUCTURE and NEWHYBRIDS (13 and 7 individuals, respectively) can be attributed to a decreased efficiency of hybrid identification when the proportion of hybrids is low, known to be more pronounced for NEWHYBRIDS than STRUCTURE [45], or to the potential confounding effect of population structure in the identification of hybrids. Lastly, the detection of hybrids is more challenging when divergence is based primarily on allele frequency differences rather than fixed alleles [90].

Microsatellite genotypes of *S*. *fuscescens* showed a bimodal distribution pattern, characterized by a dominance of parental genotypes with few intermediates or hybrids [91]. The absence of F1 hybrids and the small proportion of hybrids identified as F2 support the hypothesis of limited contemporary interbreeding between clusters. In hybrid zones, such a bimodal distribution of nuclear genotypes has been associated with strong prezygotic barriers (reviewed in [92]). In *S*. *fuscescens* where reproduction is via external fertilization in spawning aggregations [93, 94], temporal variation in spawning period among genotype clusters may represent a prezygotic reproductive barrier, although this hypothesis remains to be tested. While mate choice in spawning aggregations is unlikely, assortative mating may be caused by demographic effects, i.e. differences in the relative abundance and effective population size of the two genotype clusters in the contact zone. Of the 8 locations sampled, six locations predominantly harbored one of the two clusters (96% - 100% of the size of a sample; Table 3). Only in two locations (MAS, MOR), were both Clusters 1 and 2 well-represented.

Geometric morphometric analysis revealed subtle, yet significant variation in body shape consistent with nuclear clusters, providing further support to the recognition of two cryptic species within *S*. *fuscescens* from the northern Philippines and Hong Kong. Concordance of morphological variation with nuclear genotypes, and not mitochondrial lineages, has been observed for recently-diverged groups with potentially high levels of hybridization and mitochondrial introgression [95–97]. Moreover, shape variation may provide initial clues to ecomorphological adaptation allowing for resource partitioning and coexistence of the cryptic *S*. *fuscescens* species in coastal and seagrass habitats. In particular, variation in snout length and body depth, has been correlated with diet differences [98] and foraging in simple versus complex microhabitats [99]. Laboratory experiments [100] as well as *in situ* observations in macroalgal beds [101] have shown selective feeding in *S*. *fuscescens*. Further studies on microhabitats and diet preferences in *S*. *fuscescens* are warranted. Body shape differences may be used to test the hypothesis of potential microhabitat or diet differences between the two cryptic species here uncovered within *S*. *fuscescens*.

### Microsatellite markers provide no support to the hypothesis of reproductive isolation of mitochondrial lineage C

*Siganus fuscescens* collected from the northern part of the species' distributional range exhibited mitochondrial phylogenetic diversity with three mtDNA lineages co-occurring: Clade A, Clade B, Clade C [19, 21, 23, 26]. Despite substantial sequence divergence separating Clade C from Clades A and B (3.3% for the mitochondrial CR and 6.2% for the cyt*b* marker), with divergence times dating back to the late Pliocene-early Pleistocene (1.8–5.2 MYa) [19, 26], no matching genetic differences were shown by microsatellite markers. While Cluster 1 and Cluster 2 were associated with, respectively, Clade A and Clade B, Clade-C individuals did not flag a distinct nuclear genotype cluster. Instead, all five Clade-C individuals were found within Cluster 2. The pattern of individuals possessing a Clade-C haplotype thought to be characteristic of *S*. *canaliculatus* [19], but with an *S*. *fuscescens* nuclear background is consistent across different nuclear marker types, including ITS sequences [20], AFLP [21], and microsatellites (this study). Thus, the present study provided no support to the hypothesis that distinct mtDNA lineage C would represent a reproductively isolated lineage, at least for the *S*. *fuscescens* populations from the Philippines and Hong Kong. However, such a conclusion cannot be extended to other locations across the broader Indo-Pacific where distinct mitochondrial lineages also occur in sympatry, pending an analysis of nuclear markers.

### Mitonuclear discordance and mitochondrial introgression: Pleistocene vicariance and secondary contact in the South China Sea

Mitonuclear discordance is a common phenomenon in animal systems, particularly for recently-diverged taxa or incipient species (reviewed in [35]). For *S*. *fuscescens*, roughly a fifth of all sampled individuals exhibited mitonuclear discordance, largely due to Clade A mtDNA haplotypes occurring in both nuclear Clusters 1 and 2. Geographical differences in the spatial distribution of mtDNA lineages and nuclear clusters tend to dismiss incomplete lineage sorting as the cause for mitonuclear discordance [33, 35]. Similar geographical distributions are observed for associated mtDNA lineages and nuclear clusters, i.e. Clade A- Cluster1, Clade B-Cluster 2, but distributions are markedly different between the two groups. In particular, Cluster 1 is widely distributed across the Philippines (R. Ravago-Gotanco, unpublished data), parallel to the distribution of its associated mitochondrial Clade A, which is widespread across the Philippines [19], the Japanese archipelago, and across the Coral Triangle [21, 23]. Meanwhile, Cluster 2 has been found only in the South China Sea and north Philippine Sea basins, and not in other locations in the Philippine archipelago (R. Ravago-Gotanco, unpublished data). Its associated mitochondrial Clade B exhibits a similarly restricted distribution in the northern periphery of the distributional range of *S*. *fuscescens*, in the South China Sea and Philippine Sea basins [19, 23], and regions further north, i.e. the coast of China, the Japanese archipelago, and Taiwan [21, 23, 26], although it was also found in low frequency at two southern locations in the South China Sea and the Indian Ocean [23].

Mitochondrial introgression, and not incomplete lineage sorting, is the likely cause of the observed mitonuclear discordance, as evidenced by asymmetric mitochondrial gene flow between genotype clusters. Introgression can arise from multiple demographic, selective, and behavioural mechanisms. These include neutral drift following population or range expansion [102], asymmetry in the relative abundance of hybridizing taxa [103], sex-biased dispersal [104, 105], asymmetry in mate choice [106], and differential selection [28, 107, 108]. For *S*. *fuscescens* where reproduction occurs through external fertilization of gametes released in spawning aggregations [92, 93], mate choice, sex-biased dispersal, and other behavioural mechanisms favouring heterospecific crosses appear unlikely. A more parsimonious explanation for the mitonuclear discordance and differential geographical distribution patterns of mtDNA clades and genotype clusters is a neutral process following secondary contact of lineages diverged in allopatry. Demographic, and presumably spatial expansion of *S*. *fuscescens* Clade A and Clade B has been previously suggested [19]. In particular, Clade B is thought to have originated in areas north of the Coral Triangle, when the South China Sea and East China sea were isolated during Pleistocene glacial maxima. Clade B revealed a more pronounced genetic signature of demographic expansion estimated at ~20,000 years BP, consistent with the timing of the last Pleistocene glacial maxima, whereas Clade A underwent a more recent expansion (<10,000 years BP). The resulting demographic, and presumably southward range expansion of Clade B may have resulted in secondary contact with the ancestral Clade A which is widespread in the South China Sea and northern Philippine Sea. Asymmetric mitochondrial gene flow from Cluster 1 to Cluster 2A and from Cluster 1 to Cluster 2B, accounts for mitochondrial introgression of Clade A haplotypes into Cluster 2 individuals, resulting in a preponderance of discordant individuals with a Clade A haplotype and a Cluster 2 nuclear background. Since neutral introgression occurs primarily from the local to the invading species [101], and levels of introgression are higher for mitochondrial genes than nuclear genes [34, 109, 110], the introgressed Clade A-Cluster 2 individuals are consistent with a scenario of introgression of a local southern haplotype (Clade A) into invading Clade B-Cluster 2 individuals from expanding northern populations. The genetic signatures of Pleistocene allopatric divergence, followed by demographic expansion, secondary contact, and introgression have been similarly observed in other marine teleosts across the broader Coral Triangle region. These include the unicornfish species *Naso hexacanthus* and *N*. *caesius* [111], and four species of surgeonfishes *Acanthurus* spp. [112]. Across the northwest Pacific, two Pleistocene-diverged mitochondrial lineages of ice goby *Leucopsarion petersii* which have differential geographical distribution and phenotypic differences, exhibited mitochondrial introgression at admixture zones [113]. Extensive mitochondrial introgression was likewise reported for lineages of the three-spined stickleback *Gasterosteus aculeatus* sampled across the northern Pacific (Japan, Russia, and Alaska), with almost complete mitochondrial replacement of Japanese populations with mtDNA characteristic of Pacific lineages, despite very limited nuclear gene flow (low proportions of F1 hybrids) as estimated from allozyme markers [114]. In the South China Sea, Qiu et al. [115] reported secondary contact resulting in the occurrence of F1 hybrids between two closely-related mitochondrial lineages of the four-eyed sleeper *Bostrychus sinensis*, with lineages exhibiting a pronounced clinal latitudinal distribution. Extensive mitochondrial introgression concomitant with demographic expansion in the Pleistocene has also been inferred between two sergeant fishes, *Abudefduf sexfasciatus* and *A*. *vaigiensis* lineage A, whose geographic distributions largely overlap in the Coral Triangle [116]. These congruent results from various marine teleosts across the broader Coral Triangle, northwest Pacific and South China Sea suggest that mitochondrial introgression in recently-diverged lineages may be common, despite apparent reproductive isolation supported by limited nuclear gene flow.

Mitochondrial introgression in *S*. *fuscescens* does not appear to be recent. The low frequency of hybrids, all of which were identified as F2 and possibly even later-generation (backcrossed) hybrids, indicate that there is limited contemporary gene flow between the two clusters. Further, while putative hybrids were the most centrally located of all the 343 individuals in the correspondence analysis, they were still clearly localized within either cluster of non-overlapping individuals (2 and 5 individuals in Cluster 1 and Cluster 2, respectively). The putative hybrids were not found to be geometrically intermediate between clusters, as would be expected for F1 hybrids. This pattern of relative abundance of backcross hybrids despite the rarity or absence of F1 hybrids has been observed in several taxa [117], and is widely thought to be due to strong pre- or post-zygotic isolating mechanisms precluding the formation of F1 hybrids.

Within the contact zone, the degree of mitonuclear discordance and mitochondrial introgression varied among the 7 South China Sea populations. The HKG population had the highest level of discordance, where Clade A-Cluster 2 individuals represented 90.9% of the population and accounted for almost 30% of all discordant individuals. Four Philippine populations (CUR, MAS, MOR and PAT) exhibited moderate proportions of introgressed individuals (17.9% to 42.5%). Mitonuclear discordance was not observed in the SNF and BOL samples, which may be attributed to the relative rarity of Clade B in these areas (only 2 of 106 total individuals sampled), and the absence of Cluster 2 individuals from these locations. This interesting distribution pattern of mtDNA lineages and genotype clusters might be a consequence of the potentially restricted gene flow or isolation of the Lingayen Gulf (BOL, SNF) from the rest of northwestern Philippine coast due to hydrographic circulation patterns [19]. Habitat availability may also have influenced demographic processes and recruitment patterns leading to the relative dominance of Cluster 1 individuals in these areas [19]. However, we cannot eliminate the possibility of differential selection or adaptive divergence mechanisms accounting for the rarity of Clade-B and Cluster-2 genotypes in the Lingayen Gulf. Further studies are needed to examine these competing hypotheses of neutral versus adaptive processes shaping fine-scale genetic diversity across the western Luzon coast.

## Conclusion

This study reports the existence of two cryptic species of *S*. *fuscescens* in the northeast South China Sea (Philippines and Hong Kong) and northern Philippine Sea region, supported by the recovery of two nuclear clusters characterized by limited contemporary gene flow and concordant morphological differences. Mitonuclear discordance due to hybridization, introgression, and a complex demographic history obscures phylogenetic relationships for recently-diverged taxa such as the sibling species within the mottled rabbitfish species complex. The lack of correspondence between mitochondrial lineage Clade C and nuclear variation for individuals collected from the South China Sea strongly cautions against the use of mitochondrial markers alone for distinguishing *S*. *canaliculatus* from *S*. *fuscescens*. Similarly, because of extensive mitochondrial introgression, the mitochondrial marker is mostly helpless for distinguishing the two cryptic species here uncovered within *S*. *fuscescens*. Further studies are needed to determine the generality of these observations for other regions across the distributional range of *S*. *canaliculatus* and *S*. *fuscescens*, to help resolve the taxonomic ambiguities in this species complex. Geometric morphometric analysis proves a promising tool which can be further explored for the identification of the two cryptic species under *S*. *fuscescens*. Studies on the reproductive behavior and ecology, e.g. feeding ecology and microhabitat use are warranted to elucidate ecological partitioning and reproductive isolation between the two cryptic species.

## Supporting information

S1 Fig Multiplex-PCR fragment profiles diagnostic for mitochondrial lineages in the mottled rabbitfish species complex. Diagnostic amplicons from *S*. *fuscescens* Clade A (lanes 1–4, ~850 bp), *S*. *fuscescens* Clade B (lanes 5–8, ~450 bp and 850 bp), and Clade C (lane9, ~250, 450, 700, and 850 bp) are shown. M, 1 Kb Plus DNA Ladder (Invitrogen). Fragment sizes are indicated in basepairs (bp).
(TIF)

S2 Fig Landmarks and measurements for geometric morphometric analysis of external shape in *Siganus fuscescens*. (TIF)

S3 Fig Ordination plot of principal coordinate analysis of population pairwise genetic distances for *Siganus fuscescens* microsatellite genotypes and mtDNA sequences. (A) Scatterplot of population pairwise *F*_*ST*_ estimates based on microsatellite data. Red circles correspond to Cluster 1 individuals for each population, blue circles to Cluster 2 individuals for each population. (B) Scatterplot of population pairwise *Φ*_ST_ estimates based on mtDNA control region sequence data. Closed circles correspond to Clade A haplotypes for each population, open circles to Clade B haplotypes for each population.
(TIF)

S4 Fig Plots of standardised genetic distance versus geographic distance in *Siganus fuscescens* from the northern Philippines and Hong Kong. Pairwise genetic distance [Fst/(1-Fst)] was plotted against the Napierian logarithm of geographic distance. SD ship distance (in km), measured using the path tool of Google Earth (http://www.google.com/earth/). Closed circles correspond to comparisons between samples from within the Philippine archipelago. Open circles correspond to comparisons across the South China Sea (i.e. Philippines vs. Hong Kong). Dotted lines indicate linear regression of genetic distance against geographic distance within the Philippine archipelago. There was no evidence of increasing genetic distance with increasing geographic distance at the scale of the Philippine archipelago, except, perhaps, for Cluster2 *S*. *fuscescens*. There was no evidence of an effect of isolation by distance at the scale of the northern South China Sea except, perhaps, for mitochondrial Clade B. Population-pairwise genetic distances were calculated separately for (A) Cluster1. (B) Cluster 2. (C) Mitochondrial Clade A. (D) Mitochondrial Clade B.
(TIF)

S1 Table Mottled rabbitfish species complex: Genbank accession numbers for the mtDNA control region sequences used in this study for mitochondrial lineage identification and their frequency distribution across sampling locations. (PDF)

S2 Table Genetic diversity estimates of *Siganus fuscescens* populations and genotype clusters at 12 microsatellite loci. Estimates are calculated by grouping individuals according to location and genotype cluster. The number of individuals (N), number of alleles (Na), observed heterozygosity (H_O_), expected heterozygosity (H_E_), inbreeding coefficient (*F*_*IS*_) and its associated significance value (*P*) are presented for each sample at each locus, and across all loci. *P* values significant following a sequential Bonferroni correction are highlighted.
(PDF)

S3 Table Matrix of pairwise *F*_*ST*_ values and associated *P* values for *Siganus fuscescens* samples grouped by genotype clusters and sampling location. Below the diagonal: pairwise *F*_*ST*_ values. Above the diagonal: *P* values. Significant *P* values following the sequential Bonferroni correction are highlighted.
(PDF)
