# Supplementary figures and images for: Cryptic genetic diversity in the mottled rabbitfish Siganus fuscescens with mitochondrial introgression at a contact zone in the South China Sea
Source: PLoS One. 2018 Feb 21;13(2):e0193220. doi: 10.1371/journal.pone.0193220 (PMC5821360; doi:10.1371/journal.pone.0193220)

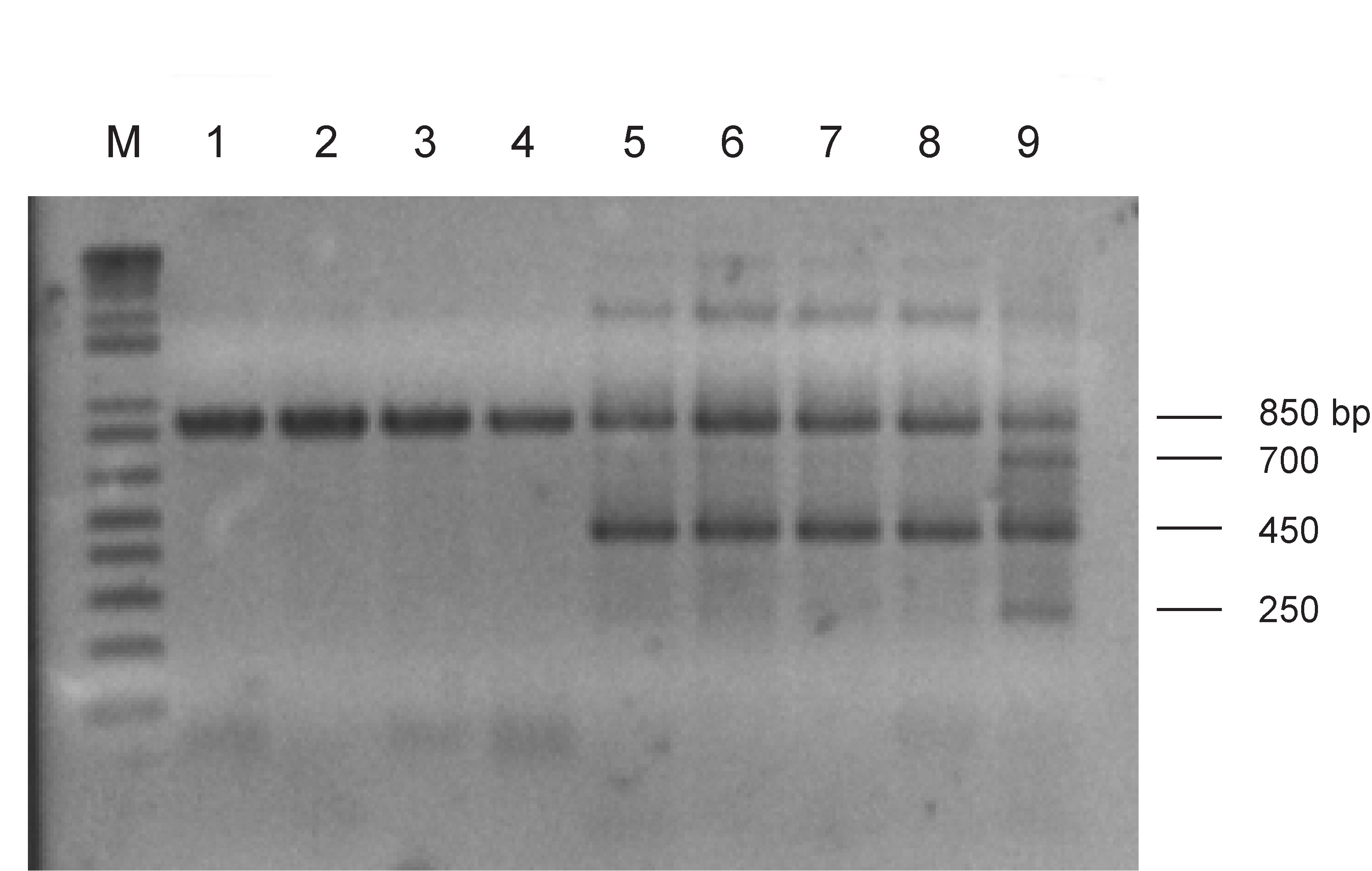

Supplement: S1 Fig — Diagnostic amplicons from S. fuscescens Clade A (lanes 1–4, ~850 bp), S. fuscescens Clade B (lanes 5–8, ~450 bp and 850 bp), and Clade C (lane9, ~250, 450, 700, and 850 bp) are shown. M, 1 Kb Plus DNA Ladder (Invitrogen). Fragment sizes are indicated in basepairs (bp). (TIF) [file pone.0193220.s001.tif]

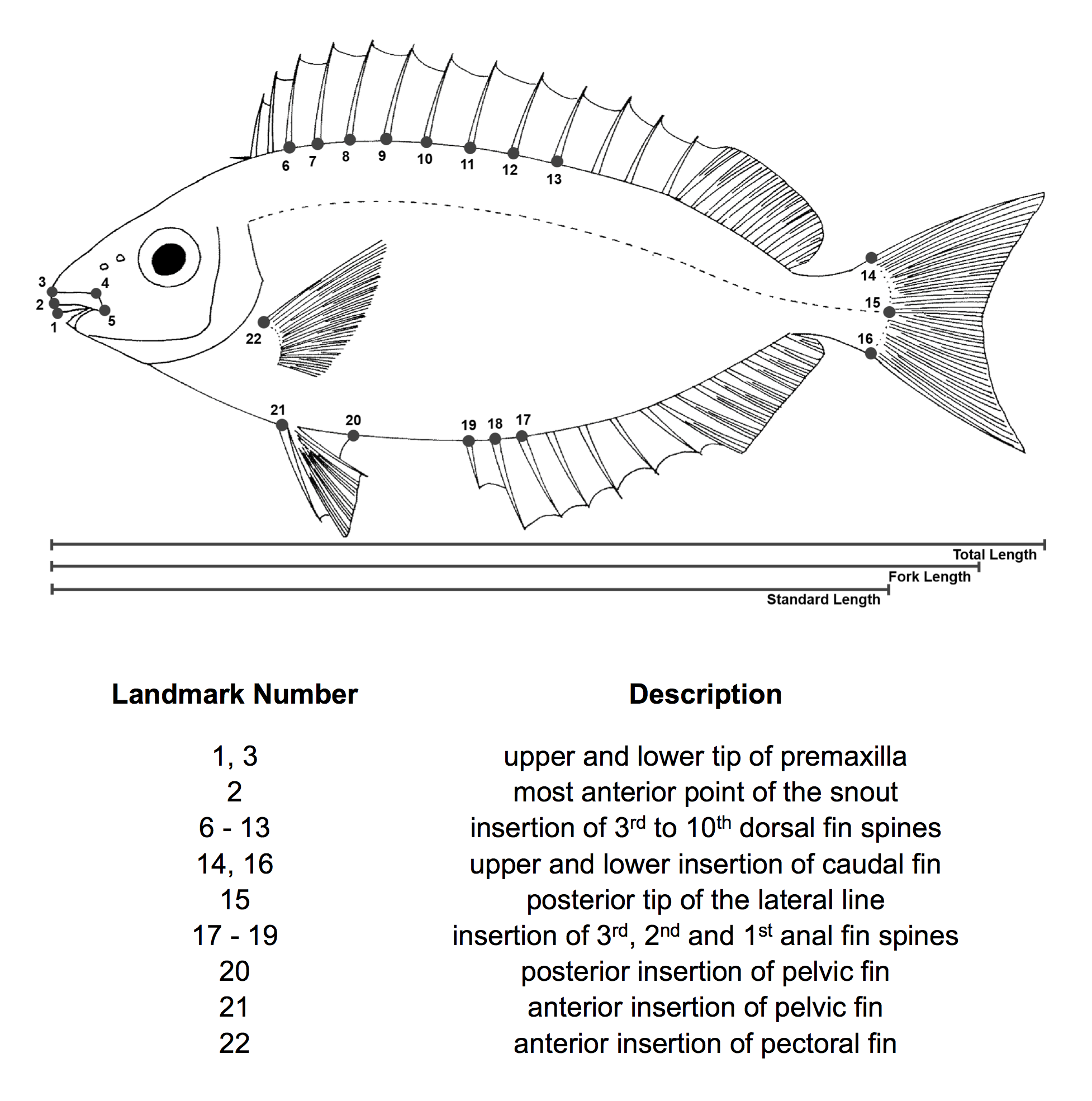

Supplement: S2 Fig — (TIF) [file pone.0193220.s002.tif]

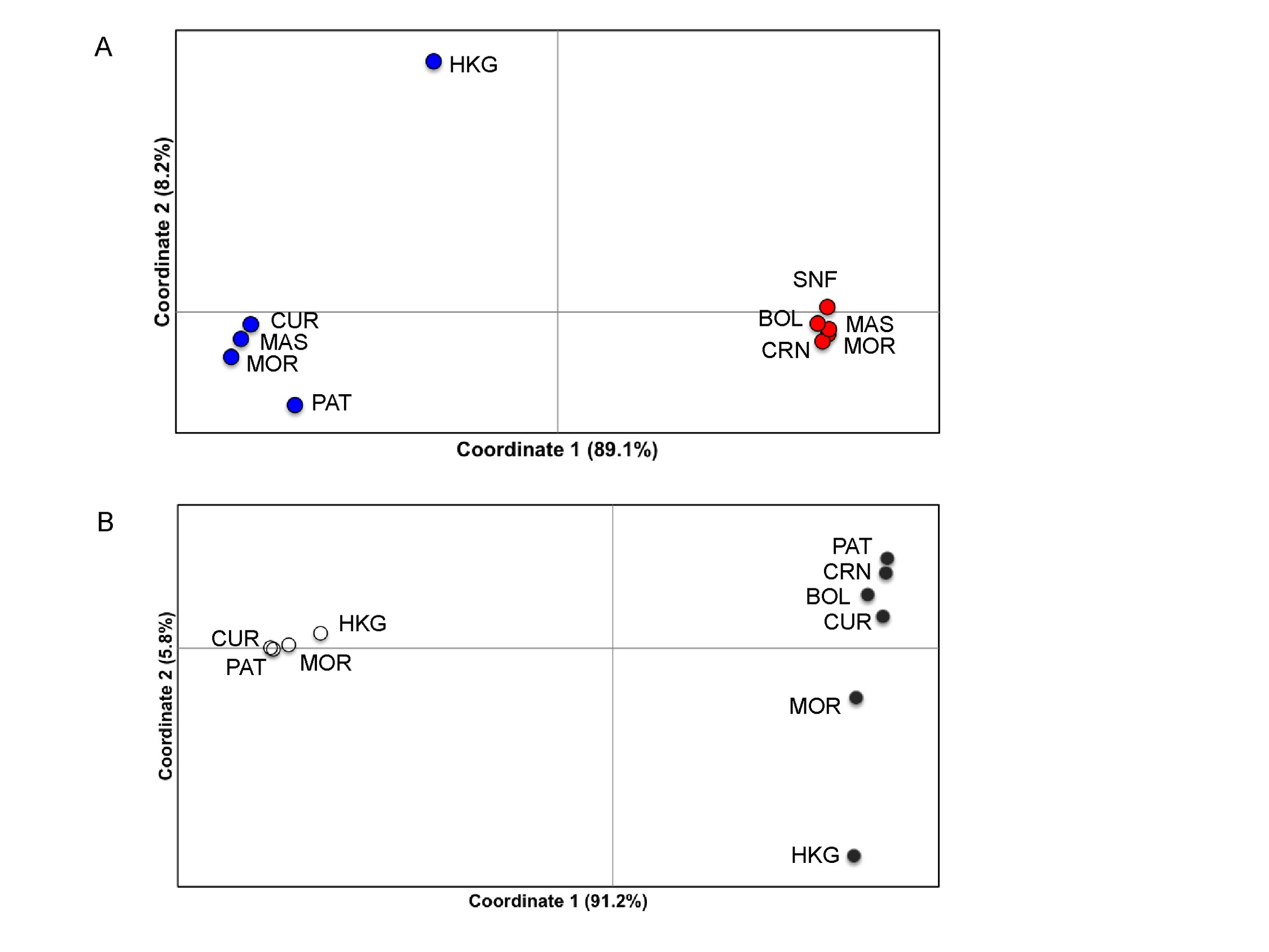

Supplement: S3 Fig — (A) Scatterplot of population pairwise FST estimates based on microsatellite data. Red circles correspond to Cluster 1 individuals for each population, blue circles to Cluster 2 individuals for each population. (B) Scatterplot of population pairwise ΦST estimates based on mtDNA control region sequence data. Closed circles correspond to Clade A haplotypes for each population, open circles to Clade B haplotypes for each population. (TIF) [file pone.0193220.s003.tif]

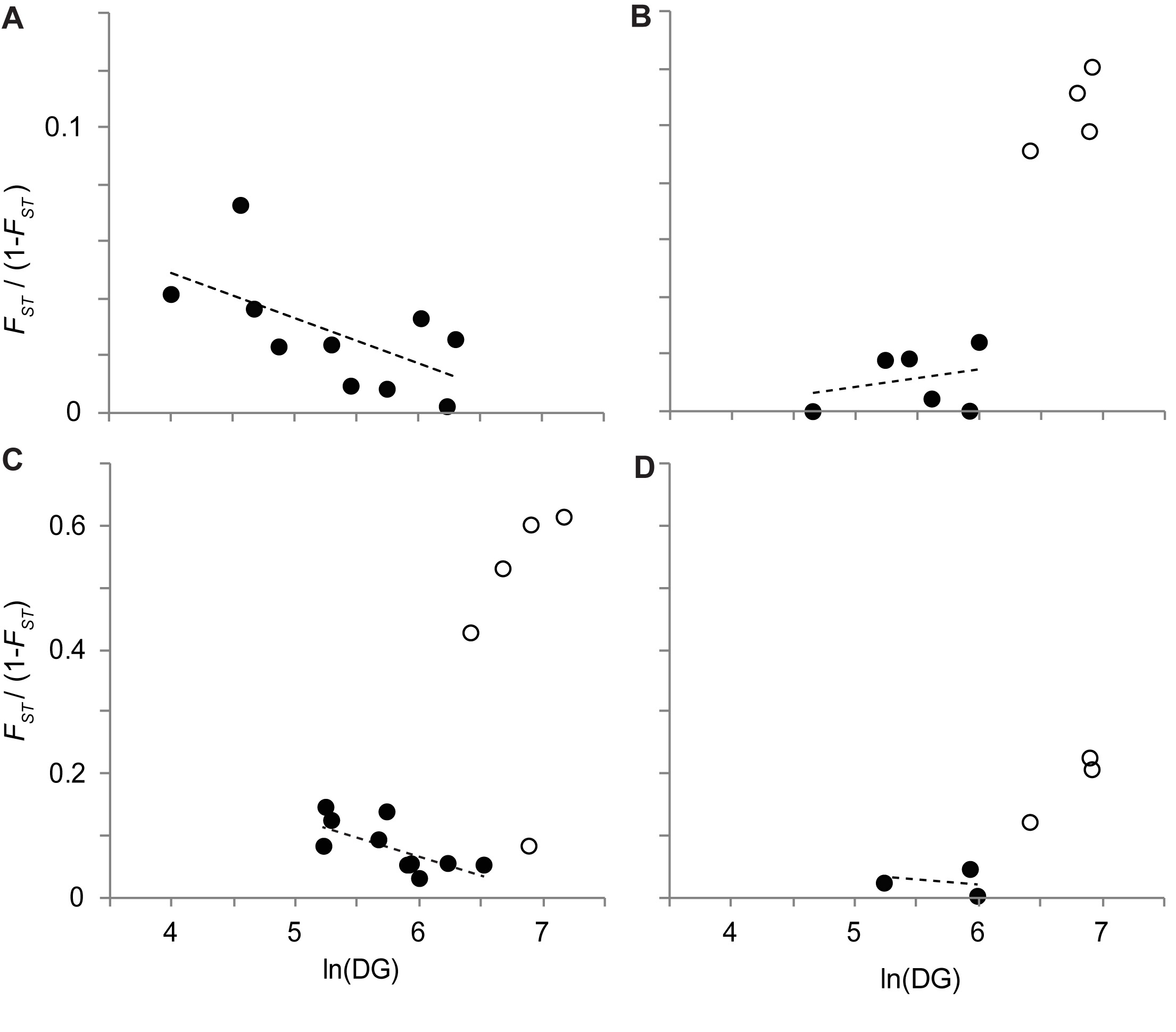

Supplement: S4 Fig — Pairwise genetic distance [Fst/(1-Fst)] was plotted against the Napierian logarithm of geographic distance. SD ship distance (in km), measured using the path tool of Google Earth (http://www.google.com/earth/). Closed circles correspond to comparisons between samples from within the Philippine archipelago. Open circles correspond to comparisons across the South China Sea (i.e. Philippines vs. Hong Kong). Dotted lines indicate linear regression of genetic distance against geographic distance within the Philippine archipelago. There was no evidence of increasing genetic distance with increasing geographic distance at the scale of the Philippine archipelago, except, perhaps, for Cluster2 S. fuscescens. There was no evidence of an effect of isolation by distance at the scale of the northern South China Sea except, perhaps, for mitochondrial Clade B. Population-pairwise genetic distances were calculated separately for (A) Cluster1. (B) Cluster 2. (C) Mitochondrial Clade A. (D) Mitochondrial Clade B. (TIF) [file pone.0193220.s004.tif]
