# Supplementary material for: Cryptic genetic diversity in the mottled rabbitfish Siganus fuscescens with mitochondrial introgression at a contact zone in the South China Sea
Source: PLoS One. 2018 Feb 21;13(2):e0193220. doi: 10.1371/journal.pone.0193220 (PMC5821360; doi:10.1371/journal.pone.0193220)
Supplement: S1 Table — (PDF) [file pone.0193220.s005.pdf]

S1 Table. Mottled rabbitfish species complex: GenBank accession numbers for the mtDNA control region sequences used in this study for mitochondrial lineage identification and their frequency distribution across sampling locations.

MtDNA Lineage	Haplotype No.	GenBank Accession	Location						
			HKG	CUR	BOL	MOR	PAT	CRN	TOTAL
Clade A	5	GU929567			1			2	3
	6	GU929570		1					1
	7	GU929573		4	1	1	3	3	12
	8	GU929574	1	1	2	1		2	7
	9	GU929575		1	10		1	3	15
	10	GU929576	11	2		5			18
	11	GU929578		1	3			6	10
	12	GU929579		1					1
	13	GU929580			1				1
	14	GU929581			1				1
	15	GU929582			1				1
	16	GU929583	1		1				2
	17	GU929584			1				1
	18	GU929585			1		1		2
	19	GU929586			1				1
	20	GU929588				1			1
	21	GU929590				1			1
	22	GU929591				1			1
	23	GU929593				1			1
	24	GU929596				1			1
	25	GU929597				1			1
	26	GU929598				1		1	2
	27	GU929600	1					1	2
	28	GU929608						1	1
	29	GU929609						1	1
	30	GU929610						1	1
	31	GU929611						1	1
	32	GU929612						1	1
	33	GU929613						1	1
	34	GU929627					1		1
	35	GU929628					1		1
	36	GU929659	1						1
	37	GU929660	1						1
	38	GU929661	1						1
	39	GU929662	1						1
	40	GU929663	1						1
	41	GU929664	1						1
	42	GU929665	1						1
	43	GU929667	1						1
	44	GU929668	1						1
	45	GU929669	1						1
Clade B	46	GU929566		1			1		2
	47	GU929568		5		2	7		14
	48	GU929572		1					1
	49	GU929577		1					1
	50	GU929589				2			2
	51	GU929592				1			1
	52	GU929593					1		1
	53	GU929594				1			1
	54	GU929595				1			1
	55	GU929622					2		2
	56	GU929623	1				2		3
	57	GU929624					1		1
	58	GU929625					2		2
	59	GU929626					1		1
	60	GU929629					1		1
	61	GU929666	1						1
Clade C	1	GU929670		1					1
	2	GU929671	1						1
	3	GU929672	1						1
	4	GU929673				1			1
**TOTAL**			**28**	**20**	**24**	**22**	**25**	**24**	**143**
