# Supplementary material for: Cryptic genetic diversity in the mottled rabbitfish Siganus fuscescens with mitochondrial introgression at a contact zone in the South China Sea
Source: PLoS One. 2018 Feb 21;13(2):e0193220. doi: 10.1371/journal.pone.0193220 (PMC5821360; doi:10.1371/journal.pone.0193220)
Supplement: S2 Table — Estimates are calculated by grouping individuals according to location and genotype cluster. The number of individuals (N), number of alleles (Na), observed heterozygosity (HO), expected heterozygosity (HE), inbreeding coefficient (FIS) and its associated significance value (P) are presented for each sample at each locus, and across all loci. P values significant following a sequential Bonferroni correction are highlighted. (PDF) [file pone.0193220.s006.pdf]

S2 Table. Genetic diversity estimates of *Siganus fuscescens* populations and genotype clusters at 12 microsatellite loci. Estimates are calculated by grouping individuals according to location and genotype cluster. The number of individuals (N), number of alleles (Na), observed heterozygosity (H_O), expected heterozygosity (H_E), inbreeding coefficient (F_{IS}) and its associated significance value (*P*) are presented for each sample at each locus, and across all loci. *P* values significant following a sequential Bonferroni correction are highlighted.

Sample		Locus											
HKG		Sfus6	Sfus8	Sfus9	Sfus21	Sfus22	Sfus56	Sfus76	Sfus95	Sfus97	Sfus98	Sfus113	Sfus167
	N	28	28	28	28	28	28	28	28	28	28	28	28
	Na	10	16	4	12	4	19	19	7	8	16	11	5
	H_O	0.679	0.964	0.393	0.643	0.536	0.750	0.750	0.429	0.750	0.750	0.643	0.643
	H_E	0.837	0.910	0.427	0.821	0.561	0.929	0.929	0.788	0.668	0.899	0.824	0.695
	F_{IS}	0.207	-0.041	0.097	0.235	0.063	0.210	0.210	0.471	-0.105	0.183	0.237	0.092
	P	0.022	0.850	0.563	0.004	0.708	0.000	0.000	0.001	0.642	0.000	0.014	0.855
CUR													
	N	39	39	39	39	39	39	39	37	39	39	39	39
	Na	9	15	5	11	6	22	22	7	11	14	16	4
	H_O	0.590	0.949	0.615	0.641	0.205	0.821	0.821	0.459	0.718	0.821	0.513	0.385
	H_E	0.755	0.897	0.571	0.712	0.214	0.911	0.911	0.549	0.805	0.866	0.818	0.389
	F_{IS}	0.231	-0.045	-0.064	0.113	0.053	0.112	0.112	0.177	0.121	0.065	0.384	0.025
	P	0.018	0.835	0.357	0.010	0.142	0.109	0.109	0.296	0.001	0.816	0.000	0.134
SNF													
	N	53	53	53	53	53	53	53	53	53	53	53	53
	Na	8	19	5	17	16	22	22	14	4	20	26	6
	H_O	0.566	0.925	0.642	0.887	0.830	0.774	0.774	0.717	0.170	0.830	0.736	0.113
	H_E	0.630	0.920	0.677	0.913	0.833	0.903	0.903	0.783	0.222	0.905	0.947	0.161
	F_{IS}	0.111	0.005	0.062	0.038	0.012	0.153	0.153	0.094	0.245	0.092	0.232	0.304
	P	0.624	0.438	0.923	0.043	0.660	0.004	0.004	0.026	0.000	0.087	0.000	0.006
BOL													
	N	56	56	56	56	56	56	56	56	56	56	56	56
	Na	6	15	5	18	17	21	21	14	2	20	24	4
	H_O	0.518	0.804	0.714	0.875	0.804	0.786	0.786	0.821	0.107	0.911	0.786	0.232
	H_E	0.633	0.898	0.677	0.916	0.842	0.898	0.898	0.830	0.101	0.911	0.929	0.210
	F_{IS}	0.191	0.114	-0.046	0.054	0.048	0.134	0.134	0.019	-0.048	0.009	0.163	-0.095
	P	0.172	0.207	0.671	0.291	0.261	0.006	0.006	0.499	1.000	0.735	0.000	1.000

Sample		Locus											
		**Sfus6**	**Sfus8**	**Sfus9**	**Sfus21**	**Sfus22**	**Sfus56**	**Sfus76**	**Sfus95**	**Sfus97**	**Sfus98**	**Sfus113**	**Sfus167**
**MAS**													
	**N**	28	30	30	30	30	28	28	30	30	30	30	30
	**Na**	7	15	5	14	12	18	18	13	7	15	27	3
	H_O	0.571	0.867	0.733	0.767	0.367	0.750	0.750	0.667	0.400	0.800	0.867	0.100
	H_E	0.805	0.884	0.753	0.867	0.724	0.912	0.912	0.750	0.567	0.881	0.932	0.509
	F_{IS}	0.285	0.039	0.053	0.161	0.529	0.215	0.215	0.166	0.309	0.111	0.085	0.814
	P	0.016	0.272	0.011	0.054	0.000	0.000	0.000	0.196	0.000	0.019	0.169	0.000
**MOR**													
	**N**	82	82	82	82	82	82	82	81	81	82	81	82
	**Na**	7	19	5	21	17	26	26	20	9	21	30	5
	H_O	0.537	0.890	0.549	0.817	0.549	0.732	0.732	0.704	0.383	0.841	0.716	0.207
	H_E	0.761	0.907	0.755	0.882	0.790	0.937	0.937	0.773	0.585	0.913	0.924	0.573
	F_{IS}	0.317	0.035	0.277	0.069	0.298	0.225	0.225	0.106	0.352	0.085	0.231	0.642
	P	0.000	0.536	0.000	0.000	0.003	0.000	0.000	0.028	0.000	0.032	0.000	0.000
**PAT**													
	**N**	30	30	30	30	30	30	30	30	30	30	30	30
	**Na**	7	14	4	8	8	13	13	5	10	13	15	4
	**Ho**	0.767	0.900	0.600	0.567	0.233	0.867	0.867	0.500	0.733	0.567	0.767	0.300
	**He**	0.747	0.906	0.653	0.651	0.353	0.874	0.874	0.571	0.839	0.841	0.812	0.389
	F_{IS}	-0.036	-0.018	0.121	0.177	0.346	-0.019	-0.019	0.166	0.112	0.330	0.086	0.112
	P	0.205	0.933	0.174	0.387	0.006	0.157	0.157	0.739	0.000	0.000	0.097	0.408
**CRN**													
	**N**	24	24	24	24	24	24	24	24	24	24	24	24
	**Na**	6	14	5	15	13	14	14	13	4	16	20	4
	H_O	0.583	0.792	0.542	0.833	0.792	0.875	0.875	0.792	0.167	0.958	0.875	0.208
	H_E	0.682	0.895	0.658	0.885	0.839	0.889	0.889	0.852	0.193	0.912	0.931	0.227
	F_{IS}	0.190	0.136	0.144	0.044	0.015	0.074	0.074	0.092	-0.046	-0.035	0.033	-0.070
	P	0.128	0.264	0.225	0.424	0.822	0.395	0.395	0.787	1.000	0.334	0.726	1.000

Sample	Locus											
	**Sfus6**	**Sfus8**	**Sfus9**	**Sfus21**	**Sfus22**	**Sfus56**	**Sfus76**	**Sfus95**	**Sfus97**	**Sfus98**	**Sfus113**	**Sfus167**
**Cluster1**												
**N**	195	197	197	197	197	195	195	197	197	197	196	197
**Na**	7	22	8	21	20	26	26	19	2	23	31	5
**H_O**	0.508	0.868	0.695	0.883	0.817	0.733	0.733	0.792	0.127	0.898	0.806	0.142
**H_E**	0.640	0.914	0.686	0.921	0.841	0.909	0.909	0.840	0.119	0.914	0.941	0.143
**F_{IS}**	0.210	0.057	-0.026	0.047	0.025	0.195	0.195	0.064	-0.023	0.022	0.151	0.008
P	0.002	0.008	0.005	0.019	0.793	0.000	0.000	0.122	0.004	0.470	0.000	0.680
**Cluster2A**												
**N**	117	117	117	117	117	117	117	114	116	117	117	117
**Na**	11	19	6	18	8	29	29	11	14	20	21	6
**H_O**	0.650	0.906	0.470	0.667	0.248	0.829	0.829	0.474	0.733	0.761	0.607	0.410
**H_E**	0.804	0.916	0.532	0.747	0.291	0.916	0.916	0.647	0.810	0.874	0.840	0.525
**F_{IS}**	0.114	0.012	0.195	0.093	0.225	0.047	0.047	0.178	0.136	0.165	0.200	0.094
P	0.000	0.982	0.000	0.003	0.000	0.456	0.456	0.000	0.000	0.000	0.000	0.019
**Cluster2B**												
**N**	29	29	29	29	29	29	29	29	29	29	29	29
**Na**	7	14	3	7	6	11	11	5	11	11	13	4
**H_O**	0.793	0.931	0.552	0.517	0.172	0.897	0.897	0.448	0.793	0.552	0.724	0.345
**H_E**	0.756	0.903	0.628	0.629	0.252	0.862	0.862	0.532	0.845	0.816	0.774	0.330
**F_{IS}**	0.240	-0.043	0.065	0.218	0.031	0.201	0.201	0.442	-0.122	0.174	0.257	0.088
P	0.003	0.897	0.690	0.011	0.748	0.003	0.003	0.000	0.565	0.000	0.014	0.877
**Mean over all loci**												
**N**	113.7	114.3	114.3	114.3	114.3	113.7	113.7	113.3	114.0	114.3	114.0	114.3
**Na**	8.333	18.333	5.667	15.333	11.333	22.000	22.000	11.667	9.000	18.000	21.7	5.000
**H_O**	0.650	0.902	0.572	0.689	0.413	0.820	0.820	0.571	0.551	0.737	0.712	0.299
**H_E**	0.734	0.911	0.615	0.766	0.461	0.896	0.896	0.673	0.591	0.868	0.852	0.333
**F_{IS}**	0.117	0.010	0.074	0.109	0.165	0.083	0.083	0.161	0.030	0.157	0.162	0.059
