# Supplementary material for: Cryptic genetic diversity in the mottled rabbitfish Siganus fuscescens with mitochondrial introgression at a contact zone in the South China Sea
Source: PLoS One. 2018 Feb 21;13(2):e0193220. doi: 10.1371/journal.pone.0193220 (PMC5821360; doi:10.1371/journal.pone.0193220)
Supplement: S3 Table — Below the diagonal: pairwise FST values. Above the diagonal: P values. Significant P values following the sequential Bonferroni correction are highlighted. (PDF) [file pone.0193220.s007.pdf]

**S3 Table. Matrix of pairwise $F_{ST}$ values and associated *P* values for *Siganus fuscescens* samples grouped by genotype clusters and sampling location.** Below the diagonal: pairwise $F_{ST}$ values. Above the diagonal: *P* values. Significant *P* values following a sequential Bonferroni correction are highlighted.

| | | Cluster 1 | | | | | Cluster 2 | | | | |
|---|---|---|---|---|---|---|---|---|---|---|---|
| | Sample | 1 | 2 | 3 | 4 | 5 | 6 | 7 | 8 | 9 | 10 |
| 1 | Cluster1_SNF | | 0.054 | 0.378 | 0.441 | 0.252 | 0.000 | 0.000 | 0.000 | 0.000 | 0.000 |
| 2 | Cluster1_BOL | 0.004 | | 0.108 | 0.189 | 0.523 | 0.000 | 0.000 | 0.000 | 0.000 | 0.000 |
| 3 | Cluster1_MAS | 0.002 | 0.007 | | 0.243 | 0.315 | 0.000 | 0.000 | 0.000 | 0.000 | 0.000 |
| 4 | Cluster1_MOR | 0.001 | 0.002 | 0.004 | | 0.766 | 0.000 | 0.000 | 0.000 | 0.000 | 0.000 |
| 5 | Cluster1_CRN | 0.003 | 0.000 | 0.003 | -0.001 | | 0.000 | 0.000 | 0.000 | 0.000 | 0.000 |
| 6 | Cluster2_HKG | 0.185 | 0.185 | 0.175 | 0.183 | 0.172 | | 0.000 | 0.000 | 0.000 | 0.000 |
| 7 | Cluster2_CUR | 0.279 | 0.274 | 0.276 | 0.277 | 0.267 | 0.084 | | 0.432 | 0.838 | 0.000 |
| 8 | Cluster2_MAS | 0.295 | 0.289 | 0.300 | 0.296 | 0.285 | 0.100 | 0.004 | | 0.685 | 0.009 |
| 9 | Cluster2_MOR | 0.285 | 0.280 | 0.285 | 0.284 | 0.275 | 0.089 | -0.001 | 0.000 | | 0.009 |
| 10 | Cluster2_PAT | 0.248 | 0.243 | 0.249 | 0.248 | 0.237 | 0.107 | 0.023 | 0.018 | 0.017 | |
